# Proteome wide association studies of LRRK2 variants identify novel causal and druggable proteins for Parkinson’s disease

**DOI:** 10.1038/s41531-023-00555-4

**Published:** 2023-07-08

**Authors:** Bridget Phillips, Daniel Western, Lihua Wang, Jigyasha Timsina, Yichen Sun, Priyanka Gorijala, Chengran Yang, Anh Do, Niko-Petteri Nykänen, Ignacio Alvarez, Miquel Aguilar, Pau Pastor, John C. Morris, Suzanne E. Schindler, Anne M. Fagan, Raquel Puerta, Pablo García-González, Itziar de Rojas, Marta Marquié, Mercè Boada, Agustin Ruiz, Joel S. Perlmutter, Dominantly Inherited Alzheimer Network (DIAN) Consortia, Laura Ibanez, Richard J. Perrin, Yun Ju Sung, Carlos Cruchaga

**Affiliations:** 1 Department of Psychiatry, Washington University School of Medicine, St. Louis, MO 63110 USA; 2 NeuroGenomics and Informatics Center, Washington University School of Medicine, St. Louis, MO 63110 USA; 3 Hope Center for Neurological Disorders, Washington University School of Medicine, St. Louis, MO 63110 USA; 4 Division of Biostatistics, Washington University, St. Louis, MO 63110 USA; 5 Memory Disorders Unit, Department of Neurology, University Hospital Mutua Terrassa, Terrassa, Spain; 6 Unit of Neurodegenerative diseases, Department of Neurology, University Hospital Germans Trias i Pujol and The Germans Trias i Pujol Research Institute (IGTP) Badalona, Barcelona, Spain; 7 Department of Neurology, Washington University School of Medicine, St. Louis, MO 63110 USA; 8 Department of Pathology and Immunology, Washington University School of Medicine, St. Louis, MO 63110 USA; 9 Ace Alzheimer Center Barcelona - Universitat Internacional de Catalunya, Barcelona, Spain; 10 Networking Research Center On Neurodegenerative Diseases (CIBERNED), Instituto de Salud Carlos III, Madrid, Spain

**Keywords:** Genomics, Biomarkers

## Abstract

Common and rare variants in the *LRRK2* locus are associated with Parkinson’s disease (PD) risk, but the downstream effects of these variants on protein levels remain unknown. We performed comprehensive proteogenomic analyses using the largest aptamer-based CSF proteomics study to date (7006 aptamers (6138 unique proteins) in 3107 individuals). The dataset comprised six different and independent cohorts (five using the SomaScan7K (ADNI, DIAN, MAP, Barcelona-1 (Pau), and Fundació ACE (Ruiz)) and the PPMI cohort using the SomaScan5K panel). We identified eleven independent SNPs in the *LRRK2* locus associated with the levels of 25 proteins as well as PD risk. Of these, only eleven proteins have been previously associated with PD risk (e.g., GRN or GPNMB). Proteome-wide association study (PWAS) analyses suggested that the levels of ten of those proteins were genetically correlated with PD risk, and seven were validated in the PPMI cohort. Mendelian randomization analyses identified GPNMB, LCT, and CD68 causal for PD and nominate one more (ITGB2). These 25 proteins were enriched for microglia-specific proteins and trafficking pathways (both lysosome and intracellular). This study not only demonstrates that protein phenome-wide association studies (PheWAS) and trans-protein quantitative trail loci (pQTL) analyses are powerful for identifying novel protein interactions in an unbiased manner, but also that *LRRK2* is linked with the regulation of PD-associated proteins that are enriched in microglial cells and specific lysosomal pathways.

## Introduction

Leucine-rich repeat kinase 2 (*LRRK2*) gene mutations can cause Mendelian Parkinson’s disease (PD). At the same time, common variant in this locus are some of the most significant PD risk variants. One of the largest PD risk genome-wide association studies (GWAS) to date^1^ found multiple independent signals in the *LRRK2* gene. Subsequent studies^2^ examined the association between the common variant (SNP) rs76904798 (chr12:40220632:C:T) with *LRRK2* RNA expression and found that the minor allele (T) was associated with increased expression levels of *LRRK2*. Like most of neurodegenerative diseases, PD is a complex disorder with several, and sometimes independently interconnected, pathways encompassing multiple proteins contributing to disease pathogenesis. Identifying those pathways, as well as the included proteins and protein interactions, is instrumental to decipher the pathobiology of the disease.

A recent study^3^ leveraged the cerebrospinal fluid (CSF) proteome to identify the causal proteins under the PD loci by performing cis-protein quantitative trait loci (pQTL) and Mendelian randomization (MR). A limitation of using cis-signals is the non-assessment of potential protein–protein interaction. We recently demonstrated that common variants in *MS4A4A* are a trans-pQTL for CSF soluble TREM2 (sTREM2) levels and are associated with Alzheimer’s disease (AD) risk^4^, indicating that MS4A4A modifies risk for AD by modifying TREM2 biology, and that MS4A4A is a potential therapeutic target. This led to several active clinical trials targeting MS4A4A to treat AD. Along the same lines, we performed unbiased multi-tissue pQTL mapping and identified hundreds of cis and trans pQTLs. These pQTLs were later used to identify causal and druggable targets for AD, PD, frontotemporal dementia (FTD), amyotrophic lateral sclerosis (ALS), and stroke^5^. Together, all these findings strongly support the use of pQTL analyses for the identification of new causal proteins, causal pathways, and potential therapeutic targets.

**Fig. 1:**
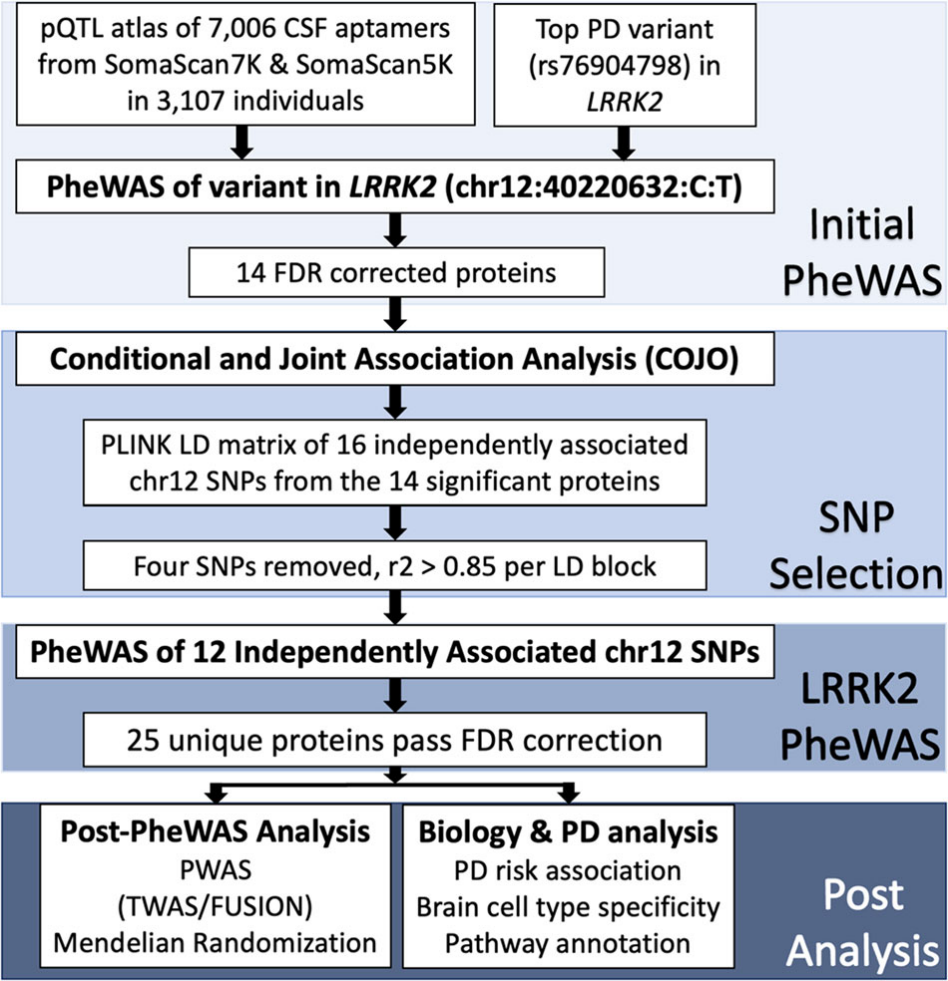
Workflow and study design. The workflow of the LRRK2 loci associated protein analysis with initial PheWAS, SNP selection, *LRRK2* PheWAS, fine mapping of significant aptamers, and analysis of proteins in terms of biological and PD significance.

Here, we performed a trans-pQTL study to identify proteins associated with *LRRK2* variants using the largest aptamer-based CSF proteomics study to date (7006 aptamers (6138 unique proteins) in 3107 individuals), and additional mediation and pathway analyses to understand the downstream effects of *LRRK2* variants (Fig. 1). We discovered that a majority of the identified proteins were enriched in the endolysosomal pathway and immune function.

## Results

### Proteins associated with *LRRK2* Parkinson’s disease risk variant

**Fig. 2:**
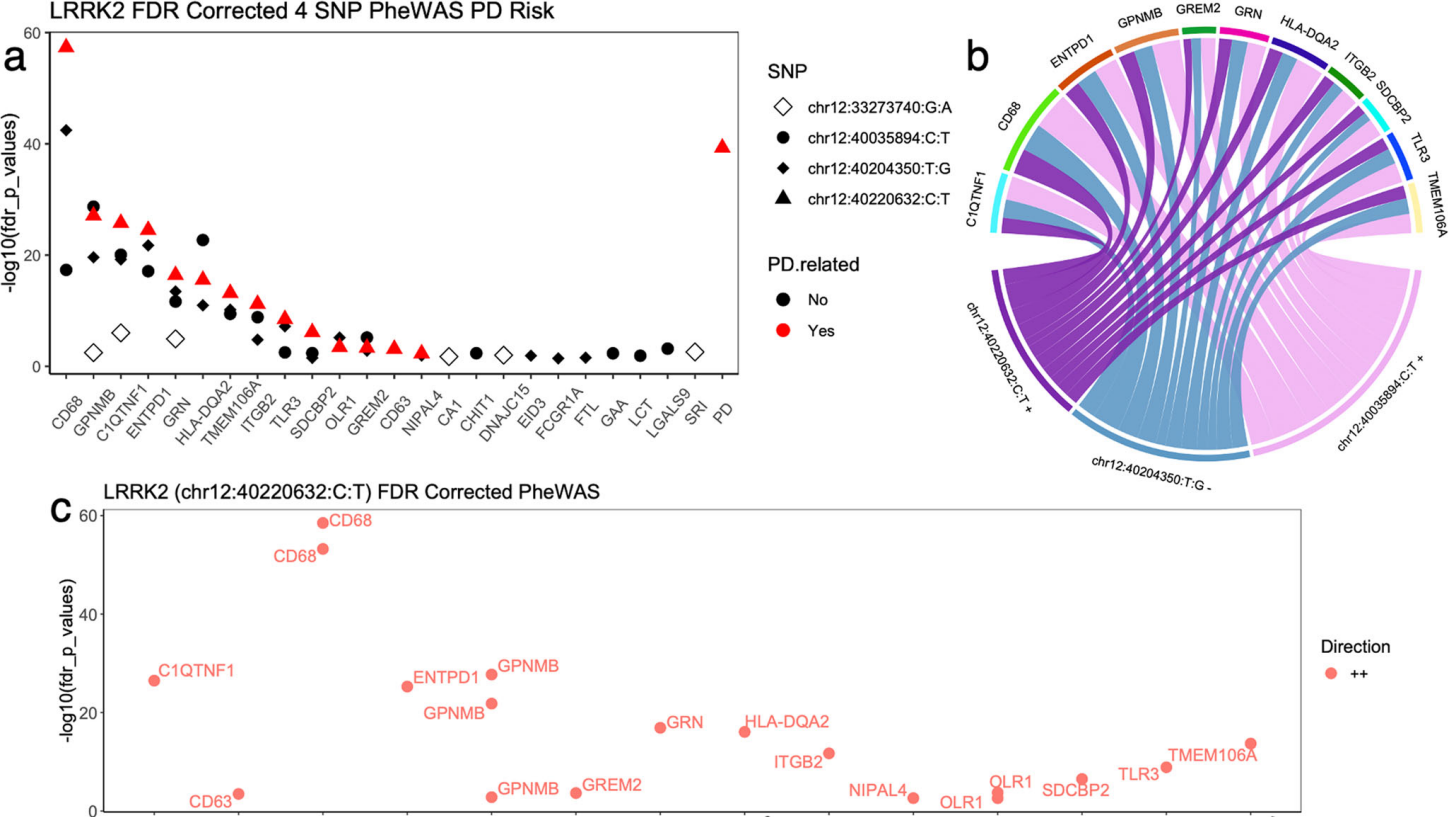
PheWAS of LRRK2 variants. **a** *LRRK2* PheWAS plot of 24 CSF proteins over four SNPs. The PD risk SNP is highlighted red. Genes previously associated with PD is shown as a color in a circle. *x* axis is proteins sorted by chr12:40220632:C:T *P* values and *y* axis is FDR corrected −log10(*P* value). For proteins with multiple aptamers, the most significant *P* value is represented. **b** Circos plot of proteins in chr12:40220632:C:T, chr12:40204350:T:G, and chr12:40035894:C:T. Width of connection represents the SNP effect size. All 14 proteins in chr12:40220632:C:T had positive effect size and the 16 proteins in chr12:40035894:C:T had a negative effect size. **c** LRRK2 PheWAS plot of 14 CSF proteins (18 aptamers). All FDR passing aptamers had positive chr12:40220632:C:T direction (light red). FDR corrected −log10(*P* value) *y* axis and *x* axis is entrez gene ID.

To identify proteins associated with *LRRK2* variants, we examined the largest CSF pQTL dataset to identify proteins associated with the sentinel variant in this locus: rs76904798. PheWAS analyses identified 14 proteins in the pQTL atlas with protein levels associated to this variant (C1QTNF1, CD63, CD68, ENTPD1, GPNMB, GREM2, GRN, HLA-DQA2, ITGB2, NIPAL4, OLR1, SDCBP2, TLR3, and TMEM106A). Then, we performed conditional analyses to identify independent signals in this region for these proteins. We identified a total of 15 SNPs associated with these proteins, and 12 SNPs were not in LD (*r*^*2*^ < 0.85). Next, we performed a PheWAS of the 12 independent SNPs and identified 11 additional proteins (AGFG2, CA1, CHIT1, DNAJC15, EID3, FCGR1A, FTL, GAA, LCT, LGALS9, and SRI) (Fig. 2a and Supplementary Fig. 1). Overall, 25 unique proteins were found to be associated with 12 independent SNPs in the *LRRK2* locus (Fig. 2b; plot made using ref. ^6^ Circlize).

Multiple proteins (such as GPNMB, CD68, OLR1, and FTL) were measured with more than one aptamer and showed high correlation (*R* > 0.9). We also analyzed the correlation accross different proteins and found that GRN and C1QTNF1 (*R* = 0.88) showed high correlation, while the remaining 20 proteins showed acorrelation of *R* < 0.7 (Supplementary Fig. 2).

Eleven (GRN, GPNMB, HLA-DQA2, CD63, CD68, EID3, ENTPD1, GAA, LCT, SRI, and TLR3) of the 25 proteins have been reported to be implicated in PD pathogenesis in previous studies (Supplementary Table 3). Six proteins (AGFG2, C1QTNF1, CHIT1, DNAJC15, FTL, and OLR1) have been reported to be implicated in other neurodegenerative diseases such as AD, ALS, or stroke, but not in PD. The rest (eight) are proteins not implicated with PD or neurodegeneration, such as GREM2, ITGB2, SDCBP2, and TMEM106A.

In order to determine if these results are robust and replicate across cohorts, we performed analyses for each of the top aptamer-SNP pairs in each of the six independent cohorts (Supplementary Fig. 3). For example, the pair GRN and chr12:40220632:C:T (MAF = 0.134) had the effect sizes of all the cohorts between 0.238 and 0.389 and were all consistently in the positive direction. The pair HLA-DQA2 and chr12:39994307:C:T (MAF = 0.049) had similar and large effect sizes between the meta pQTL analysis and the PPMI cohort with 0.725 and 0.739, respectively. We did not see any heterogeneity in any of the analyses and all the cohorts contributed to the associations. To further verify result replicability, the associations were examined in a previous study^5^. In ref. ^5^, SomaScan1.3 K (1305 proteins) was generated in 971 CSF samples, and the association of LRRK2 variants with GPNMB, GRN, OLR1, and CD63 was reported previously. These analyses indicate that the analyses are robust as all cohorts contribute to the associations and can be replicated in additional studies.

### Genetically differentially expressed proteins

Then, we integrated the protein QTL with the latest GWAS for PD risk, under the TWAS/FUSION^7^ framework to identify genetically driven differential expressed proteins in PD (Fig. 2c). This analysis identified eight proteins (HLA-DQA2, GRN, GPNMB, CD68, C1QTNF1, ENTPD1, SDCBP2, and TLR3) with differentially abundant protein levels in PD cases compared to controls, and two (ITBG2 and TMEM106A) with suggestive association (Supplementary Table 4). Higher levels in PD cases compared to controls were predicted for all proteins.

To validate these results, we used the PPMI data to determine if the levels of the PWAS-significant proteins were significantly different between controls, sporadic, PD cases, or individuals with autosomal dominant PD mutations (*LRRK2* + , *GBA* + , *SNCA* + ; prodromal cases). Of the 25 proteins (30 aptamers) identified in the initial analyses, 24 aptamers had PPMI protein data measure in 741 PPMI samples and diagnosis. We found 15 proteins had significantly different protein levels between controls and cases, controls and prodromal samples, and/or control and mutation carriers (Supplementary Fig. 4 and Supplementary Table 5), which included the ones predicted by the FUSION approach: HLA-DQA2, GRN, GPNMB, ENTPD1, ITBG2, and TMEM106A were dysregulated in PD cases, as well as even at the prodromal stages.

The PPMI data was also used to determine whether these proteins differed in expression based on sex, and 12 proteins (13 aptamers) did not have sex differences in protein expression in the PPMI dataset or when selecting only case, control, prodromal, LRRK2+, GBA+, or SNCA+ samples (Supplementary Table 6). The remaining 10 proteins (11 aptamers) had significantly different protein levels between male and female samples in PPMI. CD63, FCGR1A, a GPNMB aptamer, and a OLR1 aptamer were significant in only the case samples. GAA, LCT, and LGALS9 had significant sex differences in expression in only the control samples.

### Identifying causal proteins for PD risk

Mendelian randomization was performed to estimate the causal effect of PD exposure through links between the PD GWAS^8^ and pQTL summary statistics. Our results identified seven proteins, out of the 25, to be causally associated to PD risk (SDCBP2, GPNMB, CD68, LCT, ENTPD1, ITGB2, and C1QTNF1) (Table 1, Supplementary Table 7, and Supplementary Fig. 5). To address the horizontal pleiotropy of the *LRRK2* locus, we performed MR analyses by removing all the variants in this locus. GPNMB, CD68, and LCT remained significant. To further confirm that pleiotropy did not cause false positive findings, MR analyses were performed including only cis-pQTL, with identical results as the previous analyses. These results indicate that GPNMB, CD68, LCT, and potentially ITGB2 are part of the causal pathway for PD (Table 1).

**Table 1:** Mendelian randomization (MR) results of LRRK2-associated proteins for being causal for PD.

		All SNPs			Excluding LRRK2 (Chr12)			Cis signal		
Protein	Aptamer	# SNPs	*P* value	β	# SNPs	*P* value	β	# SNPs	*P* value	β
SDCBP2	X19261.12	1	**7.3** **×** **10**^**−21 B**^	0.562	NA	NA	NA	NA	NA	NA
GPNMB	X8606.39	1	**9.03** **×** **10**^**−20 B**^	0.201	1	**9.03** **×** **10**^**−20 B**^	0.201	1	**9.03** **×** **10**^**−20 B**^	0.201
CD68	X18922.27	4	**7.58** **×** **10**^−**11 A**^	0.219	3	**0.011**^**A**^	0.193	1	**4.01** **×** **10**^**−5 B**^	0.270
CD68	X20528.23	4	**0.001**^**A**^	0.210	3	**0.220**^**A**^	0.183	NA	NA	NA
LCT	X9017.58	3	**0.005**^**A**^	0.050	3	**0.005**^**A**^	0.050	1	**0.006**^**B**^	0.050
ENTPD1	X3182.38	2	**0.011**^**A**^	0.293	1	**0.793**^**B**^	0.022	NA	NA	NA
ITGB2	X12750.9	3	**0.022**^**A**^	0.294	2	**0.061**^**A**^	0.134	NA	NA	NA
GPNMB	X8240.207	3	**0.031**^**A**^	0.189	2	**7.99** **×** **10**^**−7 A**^	0.147	1	**9.03** **×** **10**^**−20 B**^	0.153
C1QTNF1	X6304.8	2	**0.041**^**A**^	0.293	1	0.903^B^	−0.010	NA	NA	NA
GPNMB	X5080.131	3	**0.049**^**A**^	0.166	2	**7.25** **×** **10**^**−12 A**^	0.132	1	**9.03** **×** **10**^**−20 B**^	0.135
HLA-DQA2	X7757.5	2	0.967^A^	−0.013	1	**8.27** **×** **10**^−**13 B**^	−0.195	1	**8.27** **×** **10**^**−13 B**^	−0.195
GRN	X4992.49	2	0.552^A^	0.197	1	**4.78** **×** **10**^**−5 B**^	−0.262	1	**4.78** **×** **10**^**−5 B**^	−0.262
GAA	X9385.4	1	0.141^B^	−0.112	1	0.141^B^	−0.112	1	0.141^B^	−0.112
LGALS9	X9197.4	2	0.200^A^	−0.049	2	0.200^A^	−0.049	NA	NA	NA
TLR3	X16918.198	2	0.746^A^	0.055	1	0.382^B^	−0.018	1	0.382^B^	−0.018
AGFG2	X23597.11	2	0.420^A^	0.034	2	0.420^A^	0.034	1	0.881^B^	0.007
TMEM106A	X10499.1	2	0.073^A^	0.314	1	0.436^B^	0.053	NA	NA	NA
CHIT1	X3600.2	4	0.730^A^	−0.006	4	0.730^A^	−0.006	1	0.803^B^	0.004
OLR1	X3636.37	2	0.992^A^	−4.63 × 10^−4^	1	0.793^B^	0.021	NA	NA	NA
OLR1	X7893.19	2	0.993^A^	−4.53 × 10^−4^	1	0.793^B^	0.021	NA	NA	NA
GREM2	X5598.3	2	0.115^A^	1.808	1	0.828^B^	−0.058	NA	NA	NA

^A^IVW.

^B^Wald ratio (1 SNP MR).

Number of SNPs in MR and IVW (Inverse-variance-weighted) *P* value. In total, 21 aptamers (17 proteins) had at least one SNP, in PD GWAS, with genome-wide significance (*P* < 5 × 10^−8^) in the aptamer’s pQTL. Ten aptamers were excluded from no genome-wide significant SNPs present in the PD GWAS. Wald ratio method performed for 1 IV. Effect size (β) with direction of effect. Significant *P* values are bolded. Sorted by all SNP MR *P* values, then MR *P* value without LRRK2 SNPs.

### Biological insights of the proteogenomic analyses

#### Interaction and pathway analysis

**Fig. 3:**
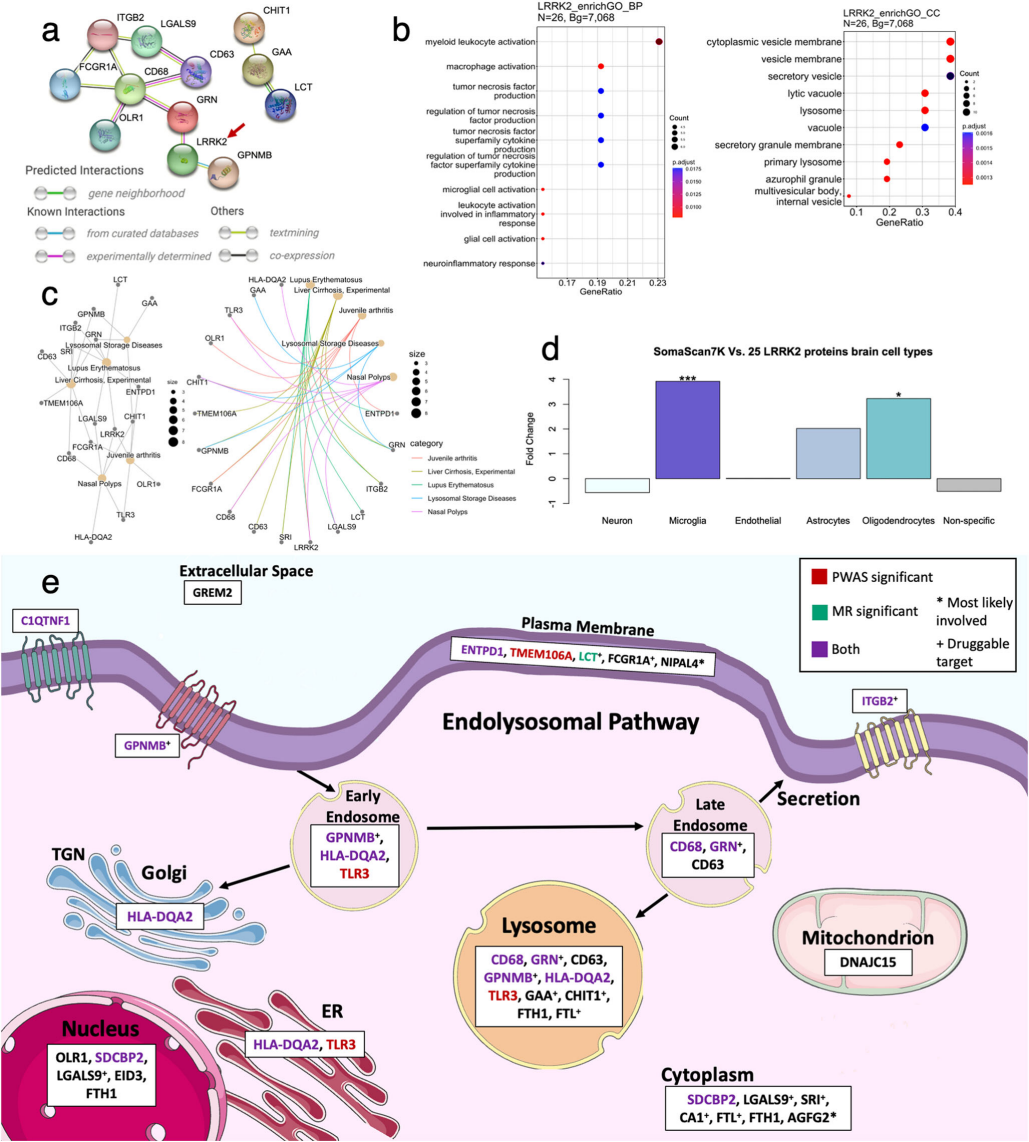
Protein pathways and cell types. **a** STRING interactions of the 25 proteins and LRRK2 (red arrow). **b** EnrichGO of 6138 SomaScan7K genes with recognized unique entrez ID as background and 26 genes of interest (25 proteins and *LRRK2*). Biological process (BP) and cellular component (CC) are shown. **c** DisGeNET gene-disease enrichment of 26 genes and 6138 SomaScan7K genes. The top five disease-gene connections are shown. **d** Brain cell-type fold-change (FC) barplot using 5709 CSF SomaScan7K proteins with brain cell-type data and 25 proteins. *** is FC > |3.7| (*P* = 3.45 × 10^−5^) and * is FC > |3.0 | . **e** Cellular pathways. Pathway involvement of the 25 LRRK2-associated proteins in the endosome, Golgi apparatus, endoplasmic reticulum (ER), nucleus, mitochondrion, cytoplasm, plasma membrane, extracellular space, and lysosome. A large proportion of LRRK2-associated proteins are involved (or enriched) in the endolysosomal pathway. *Most likely involved. ^+^Druggable protein target. Cell structure images are from Bioicons.

Pathway analysis was performed to understand how proteins interact between them, and if they were enriched in specific pathways. STRING analysis found LRRK2 to interact with GRN and GPNMB and is part of a network that also included CD68 and ITGB2 (Fig. 3a). CD68 is known to interact with GRN, CD63, and ORL1, and predicted to co-express with ITGB2 and FCGR1A. Protein enrichment analyses indicate that the 25 proteins are enriched in proteins involved in leukocyte activation involved in inflammatory response (GO:0002269, *P* = 0.008) and the microglial cell activation pathway (GO:0001774 microglial cell activation, *P* = 0.008), along with enrichment in the lysosome cellular component (GO:0005764 lysosome, *P* = 0.001; Fig. 3b). Gene-disease enrichment analyses also identified an enrichment in lysosomal storage disease genes (C0085078 Lysosomal storage disease, *P* = 0.004; Fig. 3c). Lysosomal storage disease genes include GRN, GPNMB, GAA, and CHIT1 which, according to STRING analyses, interact with CD68, CD63, and TLR3, and are known to have lysosomal membrane function (Supplementary Fig. 6).

#### Brain cell-type enrichment analysis

In line with the gene enrichment analyses, cell-type enrichment analyses indicate enrichment of microglia-specific proteins (fold change: 3.92, *P* = 3.45 × 10^−5^; Fig. 3d; Supplementary Table 8). Nine of the 25 proteins were microglial/macrophage-specific (Supplementary Table 9). Of the eleven PD-associated proteins, our analyses indicate that four (CD68, GRN, HLA-DQA2, TLR3) were microglia-specific, SRI was astrocyte-specific, and GPNMB was oligodendrocyte-specific. LRRK2, GRN, and GPNMB had predominant glial (microglia and oligodendrocyte) cell-type expression and were known to interact based on STRING (Fig. 3a).

## Discussion

Genome-wide association studies have identified over 90 loci associated with PD^8^. However, the genes driving the association for those loci and the functional mechanism by which those signals lead to PD are unknown for most of these loci. In addition, those analyses were not designed to identify complex interactions between proteins. A common approach to identify the functional and causal genes driving the association is QTL mapping combined with colocalization and Mendelian Randomization analyses. This is usually performed with transcriptomic data, but it is now understood that gene expression levels do not correlate with protein levels^5^. In fact, proteins are likely closer mechanistically to the disease outcome than gene expression. A recent study extended the eQTL approach to protein levels by analyzing pQTL for 1,305 proteins and 131 individuals from the PPMI study^5^. Kaiser et al.^3^ found that pQTLs for GPNMB, HLA-DAQ2, and FCGR2A not only colocalized with PD risk loci but were also significant in the MR analyses, implicating those proteins in the causal pathway of PD. The main limitation of this study is that they only included cis-pQTL, missing on additional causal proteins along with protein–protein interactions.

In recent studies by our group^4,5^, we used cis and trans-pQTL mapping to identify causal and druggable targets for AD and PD. We identified a highly significant trans-pQTL for TREM2 in the *MS4A4A* region that resolved the *MS4A4A* loci for AD risk^4^. We also generated a multi-tissue (brain, CSF, and plasma) pQTL atlas that was integrated with colocalization, MR, and drug repositioning to identify causal proteins and therapies for AD, PD, and other neurodegenerative diseases^5^. Yang et al.^5^ identified 35 (brain), 13 (CSF), and 15 (plasma) proteins that were PD causal based on MR with more than 50% colocalizing with PD risk loci. In addition, Yang et al.^5^ identified seven FDA compounds that target those proteins and in the same direction.

Here, we have increased the sample size of our CSF-published studies to 3107 individuals and focused on the *LRRK2* locus to resolve the most significant locus for PD risk and elucidate the downstream mechanism of *LRRK2* common variants. Briefly, we identified 25 proteins associated with PD risk, 14 of them not previously reported to be PD-associated. Subsequent TWAS/FUSION-like PWAS approaches determined that ten of those proteins are genetically associated with PD risk, and five were causally linked via MR. Among these proteins, GPNMB and HLA-DAQ2 were already identified in ref. ^3^ in their cis-only pQTL analyses. In addition, we identified other proteins (CD68 and LCT) that are causal based on all our MR analyses including stringent sensitivity analyses. Other proteins (SDCBP2, ENTPD1, ITGB2, and C1QTNF1) that our MR analyses suggest are causal were genetically associated with PD risk based on the PWAS analyses and some (ENTPD1, ITGB2, and C1QTNF1) showed significant differential protein levels in PD cases compared to controls on the PPMI dataset.

The endolysosomal pathway dysfunction is strongly implicated in the pathobiology of PD^1,3,9^. Endolysosomal system dysfunction cause disruptions in various essential cellular processes such as endocytosis, phagocytosis, macroautophagy, mitophagy, and/or lysosomal function. LRRK2 is a part of endolysosomal pathway and one of its known functions is the involvement in endosome sorting prior to lysosome fusion^10^. Interestingly, we found that LRRK2 variants modify the protein level of two well-established PD-associated proteins: GRN and GNMPB. GRN localizes in endosomes and lysosomes and GRN protein levels are known to tightly regulate lysosomal function^11^. In addition to the localization of GPNMB to plasma membrane, it is also present in intracellular vesicles such as endosomes and lysosomes^12^. Recently, GPNMB was shown to directly interact with α-synuclein, a disease-defining protein aggregating in PD^13^. Membrane protein HLA-DQA2, a protein of HLA family that has been previously implicated in PD pathogenesis^14^, also localizes in endosomes and lysosomes together with lysosome-associated proteins CD63^15^ and CD68^16^. Previously, expression of CD68 has been shown to be increased in the p.G2019S LRRK2 transgenic mice compared to control mice upon cerebral injection of α-synuclein pre-formed fibrils^17^. Importantly, the proteins identified here were not only enriched in the endolysosomal pathway, but other pathways are also related to the intracellular trafficking including the secretory vesicle pathway (GO:0099503 secretory vesicle; *P* = 0.002), or the vacuole (GO:0005773 vacuole; *P* = 0.002), indicating that changes in protein/vesicle tracking, and not just the lysosome, are important in PD pathogenesis (Fig. 3e).

In addition to identifying specific biological pathways, our analyses can also inform about the cell types in which these pathways are disrupted. We found that the proteins identified in our study were enriched in microglia (fold change: 3.92; *P* < 0.05) and immune response (*P* = 0.008). Microglia dysregulation is known to be implicated in AD, but few studies have identified a strong enrichment of microglia genes/proteins and immune function in PD^1,18^. Indeed, a large proportion of the proteins identified here play a role in the immune response. Many of these proteins are localized in plasma membrane of microglia and macrophages (such as ENTPD1 and TLR3), which also localize to lysosomes and FCGR1A, or in extracellular space (e.g., LGALS9 and CHIT1), or both (C1QTNF1, OLR1, ITGB2) (Fig. 3e). Intriguingly, many proteins (GPNMB, CD63, and HLA-DQA2) identified to be part of the endolysosomal pathway also localize to the plasma membrane and have a role in immune function^19–22^. Interestingly, a recent study found that a common noncoding variant of LRRK2 specifically contributes to risk of PD via microglial LRRK2 expression^23^. Our findings strongly support the previous observations regarding the importance of microglia and immune system function in the underlying pathobiology of PD. Independent of causality, this is the first time that all these proteins have been connected as part of the same pathways, and this helps to understand the mechanism by which *LRRK2* common variants contribute to disease pathogenesis and pathophysiology.

GRN and HLA-DQA2 had discordant effect size direction between the PWAS/FUSION and MR (Supplementary Table 4 and Table 1). Both proteins had a positive direction (higher levels in PD) for the PWAS and a negative direction for the MR analyses. PWAS/FUSION infers the levels of a protein in disease vs controls by examining the genetically regulated association between the proteome level and disease status. PWAS/FUSION is not a causal-inference method, and this prediction can be confounded by secondary events leading to disease and pleiotropy. Our PWAS results were supported by the PPMI differential protein levels analyses, in which GRN and HLA-DQA2 had increased protein levels in the PD prodromal stages and *LRRK2*^+^ cases compared to controls (Supplementary Fig. 4). Although PWAS can predict genetic association with PD risk, PWAS/FUSION does not infer causality. MR infers the potential causal effect of proteins on PD exposure through links between the PD GWAS and pQTL summary statistics. MR does not, however, infer protein levels, and PWAS is able to include that missing biological context. For the MR results, both GRN and HLA-DQA2 had negative estimates, indicating that lower levels are leading to higher PD risk. GRN having a negative estimate is in line with the known biology, as lower GRN levels are found in GRN loss of function mutation carriers^24^. Thus, our PWAS results of positive estimates agree with the PPMI observational findings and our MR results of negative estimates are in line with the known biology. Our interpretation is that PWAS predicts protein levels that can be confound by other factors and not causality. For example, increased CSF protein levels in cases could arise from higher protein release in a dysfunctional lysosome and both GRN^11^ and HLA-DQA2^14^ are involved in the endolysosomal pathway. Another example of protein levels being in the opposite direction of the causal effect is amyloid-β42 (Aβ42) in AD having higher Aβ42 levels in the brain and lower Aβ42 levels in CSF^25^. All and all, further work will be needed to address the directionality of GRN and HLA-DQA2 and the implications of discordant MR and PWAS direction results.

Finally, our study paves the way for clinical trials of new therapeutic approaches. The clinical trials targeting ITGB2 (NCT03812263), GAA (NCT00976352), and LCT (NCT02902016, NCT01145586, and NCT05100719) could be translated to PD in the future if found effective. Based on the UniProt DrugBank, there are druggable protein targets for CA1 (DB00819), CHIT1 (DB03539), FCGR1A (DB00112), FTL (DB09147), GAA (DB00284), GPNMB (DB05996), LCT (DB04779), LGALS9 (DB04472), and SRI (DB11348). Interestingly, atorvastatin^26^ and celastrol^27^ have been shown to reduce CD68 gene expression, which could similarly be applicable to PD.

In conclusion, trans-pQTL can identify protein interactions in an unbiased manner. This study linked, for the first time, LRRK2 variants with proteins that have support in our PWAS, MR, and PPMI differential protein-level analyses: some previously implicated in PD (GPNMB), and others not (C1QTNF1 and ITGB2). Our results suggest that the CSF concentrations of these proteins (such as GPNMB, C1QTNF1, and ITGB2) have the potential as biomarkers for target engagement and disease progression modification in clinical trials that target *LRRK2*.

## Methods

### Study design

We performed Phenome-wide Association Studies (PheWAS) of the sentinel SNP in LRRK2 (rs76904798) using a CSF protein QTL atlas that included more than 3000 samples to identify causal and druggable proteins for Parkinson’s disease (PD). We performed conditional analyses (GCTA-COJO) in this region, using those proteins, to identify additional independent SNPs modifying protein levels and PD risk. Next, we leveraged novel statistical approaches to identify genetically associated protein levels (Proteome-wide Association Studies (PWAS)) and causal proteins (MR). Finally, we performed pathway and cell-type enrichment analyses to determine the biological processes that these proteins are involved in, which major cell types they are expressed in, and how they interact with each other.

### Cohorts

Protein QTL was performed in a total of 3107 unrelated, Non-Hispanic White samples recruited from six different cohorts: Dominantly Inherited Alzheimer Network (DIAN), the Charles F. and Joanne Knight Alzheimer Disease Research Center (Knight ADRC), Barcelona-1, Alzheimer’s Disease Neuroimaging Initiative (ADNI), Ace Alzheimer Center Barcelona (Fundació ACE), and Parkinson’s Progression Markers Initiative (PPMI) (Supplementary Table 1). DIAN is a longitudinal study of dominantly inherited AD mutation carriers and their family members including asymptomatic and mild to moderate AD. Knight ADRC Memory and Aging Project (MAP) is a longitudinal study of cognitive functioning in persons as they age, with the goal to advance AD research. Barcelona-1 is a longitudinal observational study of dementias such as AD dementia (ADD) and mild cognitive impairment (MCI) at the University Hospital Mutua de Terrassa, Barcelona, Spain. ADNI is a multisite study that aims to develop biomarkers that will improve clinical trials for the prevention and treatment of AD. PPMI is a longitudinal observational study of participants with and without PD, with comprehensive clinical and imaging data and biological samples aimed at identifying markers of disease progression for use in clinical trials of therapies to reduce the progression of PD disability.

### Phenome-wide association study and conditional analyses

Proteins were measured using the SomaLogic aptamer-based Somascan platform. DIAN, Knight ADRC, Barcelona-1, ADNI, and Fundació ACE CSF samples were measured using SomaScan7K, which measured 7006 aptamers in 6139 protein targets. PPMI generated the CSF proteomic data using SomaScan5K, and 4785 aptamers in 4131 protein targets were measured. Genotyping and protein measurements have been described in detail elsewhere^5^. Proteomics generation was performed using the Somalogic platform in a total of 3107 unrelated non-Hispanic white individuals. Protein QC and processing followed the steps described elsewhere^28^. Initial normalization of the SomaScan7K data was performed by SomaLogic with median normalization to remove sample preparation biases and reference normalization to account for technical and biological variance. Aptamers were then removed if the aptamer’s scale factor diverged from the median scale factor >0.5 or if the median cross-plate coefficient of variation (CV) was >0.15. Aptamer values within 1.5-fold of the IQR (Interquartile range) were kept as the value. Samples and aptamers with call rate <65% were removed and the 85% call rate was applied to aptamers and samples separately after call rate recalculation. For the SomaScan5K data, scale factor and CV filtering was not performed due to the information not being available in the PPMI cohort. Using the same methods as the SomaScan7K dataset, IQR and call rate quality control was performed in the SomaScan5K dataset. Protein levels were then z-score normalized by log10-scale and values were centered to a mean around 0 and variance of 1 using the R function scale.

There were 3726 samples, with Hg38 GWAS and CSF proteomic data available, included in this study. After genetic principal component analysis (PCA) using the 1000 Genome to select European ancestry, 3328 samples remained. The 1000 Genome Project was used for ancestry grouping to select the non-Hispanic White population with gPC1 <0.005 and gPC2 >−0.01 in the PCA, and samples outside of three standard deviations from the mean were removed. After removing individuals with cryptic relatedness through identity by descent (IBD; PIHAT ≥ 0.25), 3107 samples remained. IBD was performed using certain variants (MAF ≥ 0.15 or HWE *P* ≥ 0.001, *R2* = 0.2), and one sample from each cryptic-related pair (PIHAT ≥ 0.25) was removed. pQTL linear regression was then performed using PLINK^29^ with the normalized protein levels^5^, adjusting by age, sex, the first ten principal components, and technical variables (genotyping array). For PD risk, we used the latest published GWAS, which included 2,525,897 total samples in the PD GWAS^8^.

We first determined what proteins were associated with the sentinel SNP on the LRRK2 locus (rs76904798; chr12:40220632:C:T) by performing a PheWAS for rs76904798. Multiple test correction was performed at FDR 5% correction using R package FDRestimation^30^. We then performed conditional and joint association analysis (GTCA-COJO)^31,32^ on each significant aptamer to identify independently associated SNPs (Supplementary Table 2) on this locus. SNPs with *r*^*2*^ > 0.85 per LD block were pruned to keep independently associated SNPs without non-random association. Pearson correlation was then performed using the R package corrplot^33^ to determine correlation between proteins.

pQTL mapping was performed on the six independent cohorts using PLINK^29^ with linear regression on the normalized protein levels and adjusted by the same covariates as above (age, sex, PC 1-10, and genotyping array). Signal consistency was examined in each independent dataset using forest plots of effect sizes from the linear regression analyses and compared to the effect sizes of the joint and meta pQTL analyses. I^2^ heterogeneity *P* values were gathered from the fixed-effect meta pQTL analyses performed in METAL using inverse-variance weighting per aptamer. Effect sizes of the joint analyses was then compared to the effect sizes of shared aptamers in ref. ^5^ with SomaScan 1.3K measurements from 971 CSF samples to verify replicability.

### Proteome-wide association study

A proteome-wide association study (PWAS) was performed to identify associations between PD risk and protein levels. PWAS^7^ can identify proteins potentially involved in PD etiology by examining genetically regulated association between proteome expression and PD status. Whereas these approaches were built using only cis signal expression data of the transcriptome, we modified the FUSION^7^ framework to include proteome expression weight files containing both cis and trans signals of the genes. Age, sex, the first ten genetic principal components, and genotyping array were included as covariates in the protein weight calculation model of the PWAS. The functional GWAS association statistic was calculated using the FUSION gusevlab GitHub R script^7^ and protein weights of signals on chromosome 12. FUSION post-processing was then performed to identify conditionally independent associated features by analyzing significant associations within a locus window boundary of 100,000 bp. Three strictly nonoverlapping loci were found with the second locus on the 39 to 41.5 Mb region of chromosome 12.

### Validating protein-level association with the PPMI cohort

PPMI used DNA samples from blood or saliva for genetic mutation testing of *LRRK2* (p.G2019S and p.R1441G), *GBA* (p.N370S), and *SNCA* mutations. PPMI generated the proteomic data on 1075 samples using the aptamer-based SomaScan5K platform. A total of 4783 aptamers remain after QC: outliers were removed by 1.5 IQR threshold, aptamer and individual call rate <65%, and aptamer and individual call rate <85%. There were 917 samples present with PPMI phenotypes including 185 healthy control, 545 PD, and 187 prodromal samples. Only samples with proteomic and clinical data were included in the analyses. Linear regression was performed on the normalized protein levels of PPMI using PLINK^29^ adjusted by age, sex, and cohortArray. Differential protein levels analyses were performed with control samples compared to case, prodromal, and mutation samples. Differential protein-level analyses by sex were also performed with male (*n* = 422) and female (*n* = 314) samples compared in all samples with PPMI cohort protein data and in only case, control, prodromal, and mutation samples.

### Mendelian randomization

Mendelian randomization (MR) was performed using the summary statistics of each of the significant aptamer pQTLs generated here, and the PD GWAS^8^ to investigate the potential causal effect of the proteins on PD. Using the R package TwoSampleMR^34,35^, 21 aptamers from 17 proteins had independent genome-wide significant SNPs (*P* < 5 × 10^−8^) present in the pQTL and the PD GWAS. IVW (inverse-variance-weighted) *P* values, or Wald ratio *P* values for one SNP MR, were reported. Since *LRRK2* is in a pleiotropic region and to avoid potential false positives, we performed the analyses again after removing the *LRRK2* locus (39–41.5 Mb region), on aptamers with cis signal in the MR analyses, and then performed MR on only the cis-pQTL SNP. Cis-pQTL signal was defined as the SNP being within 1 Mb upstream or downstream of the hg38 coding region of the protein-encoding gene. F-statistics were calculated for SNPs as instruments to determine the instrument strengths are at least 10 (Supplementary Table 7).

### Protein–protein interaction and pathway analysis

Pathway and interaction analyses were performed using STRING^36^, EnrichGO^37^, DisGeNET^37^, and GeneMANIA^38^. STRING was performed by entering the 25 genes that code for the proteins identified in this study together with *LRRK2* for gene interactions. EnrichGO was performed with 6138 SomaScan7K genes as background and 26 genes of interest (25 proteins and *LRRK2*). Gene-disease enrichment was performed using DisGeNET of the 26 genes and 6138 SomaScan7K genes. Network analysis was performed using GeneMANIA with the default 20 affiliated genes in a network with the 25 genes and *LRRK2*.

### Brain cell enrichment analysis

Cell-type enrichment analysis was performed on the 5706 genes in CSF SomaScan7K with cell-type data as background and the associated proteins as the target set. Brain cell types were determined using transcriptome expression data^39^ in microglial/macrophage, endothelial, mature astrocyte, neuronal, and oligodendrocyte cells isolated from human brains. Gene expression data was added for all the samples per gene, and percentiles were calculated per cell type. If one cell type had more than 50% of the sum-total expression of a gene, then that gene was considered as cell-type specific. Fold change (FC) was calculated using the ratio between cell percentage for each protein associated to *LRRK2* and the SomaScan7K background set.

## Supplementary information

Supplementary file

## Data Availability

Proteomic, pQTL, and raw data from the Knight ADRC participants are available at the NIAGADS and can be accessed at https://www.niagads.org/knight-adrc-collection. Data from the DIAN cohort is available to qualified investigators and can be requested at https://dian.wustl.edu/our-research/for-investigators/diantu-investigator-resources/dian-tu-biospecimen-request-form/. ADNI and PPMI proteomic data can be found at https://adni.loni.usc.edu/ and https://www.ppmi-info.org/, respectively.

## References

[1] Nalls MA (2019). Identification of novel risk loci, causal insights, and heritable risk for Parkinson’s disease: a meta-analysis of genome-wide association studies. Lancet Neurol..

[2] Lake J (2022). Coding and noncoding variation in LRRK2 and Parkinson’s disease risk. Mov. Disord..

[3] Kaiser S (2023). A proteogenomic view of Parkinson’s disease causality and heterogeneity. NPJ Parkinsons Dis..

[4] Deming Y, Filipello F, Cignarella F (2019). The MS4A gene cluster is a key modulator of soluble TREM2 and Alzheimer’s disease risk. Sci. Transl. Med..

[5] Yang C (2021). Genomic atlas of the proteome from brain, CSF and plasma prioritizes proteins implicated in neurological disorders. Nat. Neurosci..

[6] Gu Z, Gu L, Eils R, Schlesner M, Brors B (2014). circlize implements and enhances circular visualization in R. Bioinformatics.

[7] Gusev A (2016). Integrative approaches for large-scale transcriptome-wide association studies. Nat. Genet..

[8] Kim, J. J. et al. Multi-ancestry genome-wide meta-analysis in Parkinson’s disease. Preprint at https://www.medrxiv.org/content/10.1101/2022.08.04.22278432v1 (2022).

[9] Vidyadhara DJ, Lee JE, Chandra SS (2019). Role of the endolysosomal system in Parkinson’s disease. J. Neurochem..

[10] Schreij (2015). LRRK2 localizes to endosomes and interacts with clathrin-light chains to limit Rac1 activation. EMBO Rep..

[11] Kao AW, McKay A, Singh PP, Brunet A, Huang EJ (2017). Progranulin, lysosomal regulation and neurodegenerative disease. Nat. Rev. Neurosci..

[12] van der Lienden, M. J. C., Gaspar, P., Boot, R. Aerts, J. M. F. G. & van Eijk, M. Glycoprotein non-metastatic protein B: an emerging biomarker for lysosomal dysfunction in macrophages. *Int. J. Mol. Sci.* **20**, 66 (2018). 10.3390/ijms20010066 PMC6337583 30586924

[13] Diaz-Ortiz ME (2022). GPNMB confers risk for Parkinson’s disease through interaction with α-synuclein. Science.

[14] Naito T (2021). Trans-ethnic fine-mapping of the major histocompatibility complex region linked to Parkinson’s Disease. Mov. Disord..

[15] Fukuda M. Lysosomal membrane glycoproteins. Structure, biosynthesis, and intracellular trafficking. *J. Biol. Chem.* **266**, 21327–21330 (1991). 1939168

[16] Holness CL, Simmons DL (1993). Molecular cloning of CD68, a human macrophage marker related to lysosomal glycoproteins. Blood.

[17] Bieri G (2019). LRRK2 modifies α-syn pathology and spread in mouse models and human neurons. Acta Neuropathol..

[18] Tan EK (2020). Parkinson disease and the immune system -associations, mechanisms and therapeutics. Nat. Rev. Neurol..

[19] Saade M, Araujo de Souza G, Scavone C, Kinoshita PF (2021). The role of GPNMB in inflammation. Front. Immunol..

[20] Petersen SH (2011). The role of tetraspanin CD63 in antigen presentation via MHC class II. Eur. J. Immunol..

[21] Rudy GB, Lew AM (1997). The nonpolymorphic MHC class II isotype, HLA-DQA2, is expressed on the surface of B lymphoblastoid cells. J. Immunol..

[22] Houser MC (2022). Progranulin loss results in sex-dependent dysregulation of the peripheral and central immune system. Front. Immunol..

[23] Langston RG (2022). Association of a common genetic variant with Parkinson’s disease is mediated by microglia. Sci. Transl. Med..

[24] Meeter LH (2016). Progranulin levels in plasma and cerebrospinal fluid in granulin mutation carriers. Dement. Geriatr. Cogn. Dis. Extra.

[25] Sturchio A (2022). High soluble amyloid-β42 predicts normal cognition in amyloid-positive individuals with Alzheimer’s disease-causing mutations. J. Alzheimers Dis..

[26] Llaverias G (2004). Atorvastatin reduces CD68, FABP4, and HBP expression in oxLDL-treated human macrophages. Biochem. Biophys. Res. Commun..

[27] Cascão R (2015). Decrease of CD68 synovial macrophages in celastrol treated arthritic rats. PLoS ONE.

[28] Timsina J (2022). Comparative analysis of Alzheimer’s disease Cerebrospinal fluid biomarkers measurement by multiplex SOMAscan platform and immunoassay-based approach. J. Alzheimers Dis..

[29] Purcell S (2007). PLINK: a toolset for whole-genome association and population-based linkage analysis. Am. J. Hum. Genet..

[30] Murray MH, Blume JD (2021). FDRestimation: flexible false discovery rate computation in R. F1000Research..

[31] Yang J (2012). Conditional and joint multiple-SNP analysis of GWAS summary statistics identifies additional variants influencing complex traits. Nat. Genet..

[32] Yang J, Lee SH, Goddard ME, Visscher PM (2011). GCTA: a tool for genome-wide complex trait analysis. Am. J. Hum. Genet.

[33] Wei, T. & Simko, V. R package ‘corrplot’: visualization of a Correlation Matrix. (Version 0.92). https://github.com/taiyun/corrplot. (2021).

[34] Hemani G (2018). The MR-Base Collaboration. The MR-Base platform supports systematic causal inference across the human phenome. eLife.

[35] Hemani, G., Tilling, K. & Davey Smith, G. Orienting the causal relationship between imprecisely measured traits using GWAS summary data. *PLoS Genet.* **13**, e1007081 (2017). 10.1371/journal.pgen.1007081 PMC5711033 29149188

[36] Warde-Farley D (2010). The GeneMANIA prediction server: biological network integration for gene prioritization and predicting gene function. Nucleic Acids Res..

[37] Yu G, Wang L, Han Y, He Q (2012). clusterProfiler: an R package for comparing biological themes among gene clusters. OMICS: A J. Integr. Biol..

[38] Szklarczyk D (2021). The STRING database in 2021: customizable protein-protein networks, and functional characterization of user-uploaded gene/measurement sets. Nucleic Acids Res..

[39] Zhang Y (2016). Purification and characterization of progenitor and mature human astrocytes reveals transcriptional and functional differences with mouse. Neuron.

