# Supplementary material for: Proteome wide association studies of LRRK2 variants identify novel causal and druggable proteins for Parkinson’s disease
Source: NPJ Parkinsons Dis. 2023 Jul 8;9:107. doi: 10.1038/s41531-023-00555-4 (PMC10329646; doi:10.1038/s41531-023-00555-4)
Supplement: Supplementary file 1 — Supplementary file [file 41531_2023_555_MOESM1_ESM.pdf]

Proteome Wide Association Studies of LRRK2 variants identify novel causal and druggable proteins for Parkinson's disease

Bridget Phillips[1,2,3], Daniel Western[1,2,3], Lihua Wang[1,2,3], Jigyasha Timsina[1,2,3], Yichen Sun[1,2,3], Priyanka Gorijala[1,2,3], Chengran Yang[1,2,3], Anh Do[1,2,3,4], Niko-Petteri Nykänen[1,2,3], Ignacio Alvarez[5], Miquel Aguilar[5], Pau Pastor[6], John C. Morris[3,7,8], Suzanne E. Schindler[7], Anne M. Fagan[3,7], Raquel Puerta[9,10], Pablo García-González[9,10], Itziar de Rojas[9,10], Marta Marquié[9,10], Mercè Boada[9,10], Agustin Ruiz[9,10], Joel S. Perlmutter[3,7], Dominantly Inherited Alzheimer Network (DIAN) Consortia, Laura Ibanez[1,2,7], Richard J. Perrin[3,7,8], Yun Ju Sung[1,2,3,4], Carlos Cruchaga[1,2,3]

Author affiliations:
1 Department of Psychiatry, Washington University School of Medicine, St. Louis, MO 63110, USA
2 NeuroGenomics and Informatics Center, Washington University School of Medicine, St. Louis, MO 63110, USA
3 Hope Center for Neurological Disorders, Washington University School of Medicine, St. Louis, MO 63110, USA
4 Division of Biostatistics, Washington University
5 Memory Disorders Unit, Department of Neurology, University Hospital Mutua Terrassa, Terrassa, Spain
6 Unit of Neurodegenerative diseases, Department of Neurology, University Hospital Germans Trias i Pujol and The Germans Trias i Pujol Research Institute (IGTP) Badalona, Barcelona, Spain
7 Department of Neurology, Washington University School of Medicine, St. Louis, MO 63110, USA
8 Department of Pathology and Immunology, Washington University School of Medicine, St. Louis, MO 63110, USA
9 Ace Alzheimer Center Barcelona - Universitat Internacional de Catalunya, Barcelona, Spain
10 Networking Research Center On Neurodegenerative Diseases (CIBERNED), Instituto de Salud Carlos III, Madrid, Spain

Correspondence to: Carlos Cruchaga, PhD
Full address: 4444 Forest Park Ave, Washington University School of Medicine, St. Louis, MO 63110, USA
E-mail: cruchagac@wustl.edu
Running title: Protein PheWAS of LRRK2 variants

Keywords: Parkinson's disease; LRRK2; Proteomics; PheWAS; GRN

Supplementary Table 1 Basic demographics of the cohorts included in the study
Supplementary Table 2 Annotation of the variants associated with protein levels on the LRRK2 locus
Supplementary Table 3 Parkinson's disease risk table of LRRK2 associated proteins
Supplementary Table 4 PWAS p-values and z-scores
Supplementary Table 5 PPMI cohort validation
Supplementary Table 6 Sex differences in protein expression using PPMI cohort
Supplementary Table 7 SNPs in MR
Supplementary Table 8 Brain cell types of SomaScan7K and LRRK2 associated proteins
Supplementary Table 9 Brain cell types of LRRK2 associated proteins
Supplementary references

Supplement 2: Dominantly Inherited Alzheimer Network (DIAN) consortia investigators and coordinators

Supplementary Figure 1: PheWAS and LD matrix of LRRK2 variants
Supplementary Figure 2: Protein-protein correlation
Supplementary Figure 3: Forest plots of the independent datasets.
Supplementary Figure 4: PPMI significant TWAS Violin plots
Supplementary Figure 5: Representative MR plots.
Supplementary Figure 6: Interaction pathways of LRRK2 associated proteins

Supplementary Table 1 Basic demographics of the cohorts included in the study

Cohort	# Samples	Avg. Age (SD)	% Male
ADNI	689	73.7 (7.5)	58.2
DIAN	193	38.6 (10.7)	48.4
MAP	805	71.4 (8.7)	46.7
Barcelona-1	197	68.8 (7.5)	52.3
Fundació ACE	438	71.9 (8.3)	41.1
PPMI	785	61.8 (9.4)	55.1

Sample sizes of each of the six cohorts included (3,107 total samples). The three discovery cohorts are DIAN, MAP, and Pau. The three replication cohorts are ADNI, Fundació ACE, and PPMI. PPMI was the only cohort measured using SomaScan5K and the other five cohorts were measured using SomaScan7K.

ADNI = Alzheimer's Disease Neuroimaging Initiative; DIAN = Dominantly Inherited Alzheimer Network; MAP = Memory and Aging Project; Barcelona-1 = Hospital Sant Pau; PPMI = Parkinson’s Progression Markers Initiative.

Supplementary Table 2 Annotation of the variants associated with protein levels on the LRRK2 locus

Variant	RSID	Type	Gene : Consequence	MAF	PD SNP r^2
chr12:10160315:G:A	rs3816844	Non-coding	*OLR1* : Intron Variant	0.495	1.11×10^{-4}
chr12:10178088:G:T	rs113181367	Non-coding	*TMEM52B* : Intron Variant	0.075	3.35×10^{-4}
chr12:33273740:G:A	rs529679490	N/A	None	0.006	1.57×10^{-6}
chr12:39994307:C:T	rs140722239	Non-coding	*SLC2A13*: Intron Variant	0.047	0.030
chr12:40035894:C:T	rs11564199	Non-coding	*SLC2A13*: Intron Variant	0.047	0.031
chr12:40039522:G:A	rs78131768	Non-coding	*SLC2A13* : Intron Variant	0.047	0.031
chr12:40189191:A:G	rs2263418	Non-coding	*LRRK2-DT* : Non-Coding Transcript Variant	0.105	0.472
chr12:40194013:T:C	rs11175546	Non-coding	*LRRK2-DT* : Non-Coding Transcript Variant	0.046	0.291
chr12:40198262:T:G	rs79410089	N/A	None	0.080	0.362
chr12:40204350:T:G	rs11564273	N/A	None	0.069	0.435
**chr12:40220632:C:T**	**rs76904798**	**Non-coding**	***LRRK2* : Intron variant**	**0.134**	**1.0**
chr12:40259708:G:A	rs28903073	Non-coding	*LRRK2* : Intron variant	0.032	0.005
chr12:40320097:T:C	rs35303786	Exonic	*LRRK2* : Missense Variant	0.021	0.003
chr12:40342447:C:T	rs919174	Non-coding	*LRRK2* : Intron Variant	0.131	0.171
chr12:40397605:G:A	rs117929583	Non-coding	*MUC19* : Intron Variant	0.024	0.110
chr12:41349642:C:T	rs190000583	Non-coding	*PDZRN4* : Intron Variant	0.036	0.003

Variant name, Rs-id, and intron/exon type of the 16 independently associated chromosome 12 SNPs ordered by bp position. Only rs35303786 (chr12:40320097:T:C) is known to be exonic. Top PD and CSF *GRN* SNP rs76904798 (chr12:40220632:C:T) is shown in bold.

Supplementary Table 3 Parkinson's disease risk table of LRRK2 associated proteins

Gene	PD	Neurodegeneration	Function/Expression	Experiment Type	Citation
CD63	**Yes**	Yes	Decreased in PD	Multiplex immunoassay of circulating small EVs (sEVs)	[Picca et al., 2020]
ENTPD1	**Yes**	Yes	Decreased in PD	RT-qPCR of substantia nigra mRNA	[Garcia-Esparcia et al., 2015]
GRN	**Yes**	Yes	Decreased in PD	Meta-analysis of PD GWAS	[Nalls et al., 2019]
HLA-DQA2	**Yes**	Yes	HLA region PD association	Haplotype analysis and step-wise conditional analysis	[Hill-Burns et al., 2011]
GAA	**Yes**	Yes	Increased in PD	Lysosomal enzymatic activity assay	[Alcalay et al., 2018]
GPNMB	**Yes**	Yes	Increased in sporadic PD	Induction of lipidopathy and immunohistochemical staining	[Moloney et al., 2018]
SRI	**Yes**	Yes	Increased in PD	Immunoprecipitation and cell lines	[Genovese et al., 2020]
LCT	**Yes**	Yes	Increased in PD	Two-sample Mendelian Randomization	[Domenighetti et al, 2022]
CD68	**Yes**	Yes	*LRRK2* Expression	Human and mouse cell culture Immunohistochemistry	[Xu et al., 2020] Preprint
EID3	**Yes**	Yes	PD candidate gene	Differential expression analysis and PPI networks	[George et al., 2019]
TLR3	**Yes**	Yes	Reduced risk EOPD	SNP genotyping and odds ratios analysis	[Wang et al., 2020]
C1QTNF1	Related	Yes	Downregulated in PD patients	PD-patient specific dopaminergic cultures DE analysis	[Momcilovic et al., 2016]
DNAJC15	Related	Yes	OGC pesticide exposure PD DML	Genome-scale methylation profiling	[Go et al., 2020]
AGFG2	No	Yes	AD pathology	Differential gene expression (DGE) analyses	[Fernandez et al., 2022]
ITGB2	No	Yes	Aging and neurodegeneration	Human aging and neurodegenerative disease microarray datasets	[Mukherjee et al., 2019]
OLR1	No	Yes	Neuroinflammatory gene	Single-nuclei sequencing	[Agarwal et al., 2020]
SDCBP2	No	Yes	Neurexin protein interactor	Protein–protein interaction network and co-expression analysis	[Cuttler et al., 2021]
GREM2	No	Yes	Neuroprotection by downregulating *GREM2* genes	Microarray expression profiling	[Forcella et al., 2020]
CHIT1	No	Yes	Microglia/macrophage activation in ALS	LC-MS/MS CSF proteomics	[Karayel et al., 2022]
FTL	No	Yes	Neurodegeneration, brain iron accumulation	Postmortem brain iron histochemistry, mtDNA variant analysis	[Kurzawa-Akanbi et al., 2021]
LGALS9	No	Related	Enhances microglial TNF production	Glial culture immunocytochemistry and cytokine measurement	[Steelman et al., 2014]
CA1	No	Related	Neuropathic pain	Carbonic anhydrase inhibition and Ischaemic brain damage	[Dettori et al., 2021]
TMEM106A	No	No	Increased in AD, *TMEM106B* paralog	qPCR, western blot, and immunohistochemistry	[Zhao et al., 2021]
NIPAL4	No	No	Congenital Ichthyosiform Erythroderma	Mutational screening and sequence pathogenicity prediction	[Laadhar et al., 2020]
FCGR1A	No	No	Leptomeningeal Metastasis Biomarker	LC-MS/MS CSF proteomics and protein microarray analysis	[Juanes-Velasco et al., 2022]

Proteins are organized by having prior studies involving PD risk and/or neurodegeneration, such as AD and ALS, and neurological disorders such as stroke. The columns are the protein name, whether the protein has been shown to be associated with PD, whether there has been an article on the protein being involved in neurodegeneration, the function/effect of the protein in the chosen article, types of experiments done in the article to prove association, and the article's citation. The 11 proteins with predicted PD risk gene association are shown in bold.

Supplementary Table 4 PWAS p-values and z-scores

Protein	Aptamer	PWAS.P-value	PWAS.Z	Direction	Model	R2 Estimate	R2 P-value
HLA-DQA2	X7757.5	**2.61×10^{-47}**	14.44715	+	lasso	0.071	3.70×10^{-51}
ITGB2	X12750.9	**4.49×10^{-47}**	14.40981	+	lasso	0.033	2.80×10^{-24}
C1QTNF1	X6304.8	**6.96×10^{-46}**	14.21923	+	enet	0.081	1.40×10^{-58}
GRN	X4992.49	**4.32×10^{-42}**	13.59439	+	enet	0.057	2.70×10^{-41}
GPNMB	X8240.207	**2.32×10^{-41}**	13.47087	+	enet	0.091	4.50×10^{-66}
GPNMB	X5080.131	**1.48×10^{-38}**	12.98536	+	enet	0.067	1.20×10^{-48}
ENTPD1	X3182.38	**1.59×10^{-34}**	12.25466	+	enet	0.074	2.90×10^{-53}
TMEM106A	X10499.1	**1.51×10^{-32}**	11.87987	+	enet	0.046	1.80×10^{-33}
CD68	X20528.23	**3.66×10^{-25}**	10.36289	+	enet	0.140	1.60×10^{-78}
CD68	X18922.27	**6.83×10^{-24}**	10.07916	+	lasso	0.160	1.50×10^{-86}
SDCBP2	X19261.12	**2.88×10^{-18}**	8.71598	+	enet	0.025	2.20×10^{-14}
TLR3	X16918.198	**1.35×10^{-15}**	7.98963	+	enet	0.036	5.50×10^{-20}
OLR1	X3636.37	0.576	-0.55992	-	lasso	0.027	1.40×10^{-20}
OLR1	X7893.19	0.717	-0.36238	-	lasso	0.017	1.80×10^{-13}
AGFG2	X23597.11	NA	NA	NA	NA	NA	NA
CA1	X4969.2	NA	NA	NA	NA	NA	NA
CD63	X9190.7	NA	NA	NA	NA	NA	NA
CHIT1	X3600.2	NA	NA	NA	NA	NA	NA
DNAJC15	X7197.2	NA	NA	NA	NA	NA	NA
EID3	X8079.39	NA	NA	NA	NA	NA	NA
FCGR1A	X3312.64	NA	NA	NA	NA	NA	NA
FTL	X5934.1	NA	NA	NA	NA	NA	NA
FTL	X15324.58	NA	NA	NA	NA	NA	NA
GAA	X9385.4	NA	NA	NA	NA	NA	NA
GPNMB	X8606.39	NA	NA	NA	NA	NA	NA
GREM2	X5598.3	NA	NA	NA	NA	NA	NA
LCT	X9017.58	NA	NA	NA	NA	NA	NA
LGALS9	X9197.4	NA	NA	NA	NA	NA	NA
NIPAL4	X12864.9	NA	NA	NA	NA	NA	NA
SRI	X12356.65	NA	NA	NA	NA	NA	NA

PWAS top hits from TWAS/FUSION. 12 aptamers (10 proteins) (bolded) had significant PWAS p-values. Sorted by PWAS p-values. PWAS p-value as NA refers to TWAS/FUSION excluding the gene from the analysis due to the aptamer's SNP-heritability p-value < 0.05. The model column shows the best performing prediction model that TWAS used, R2 estimate is the cross-validation R2 of the model, and R2 p-value shows the cross-validation R2 of the model for each aptamer

Supplementary Table 5 PPMI cohort validation

Protein	FDR SNP Direction	PWAS p-value	PPMI Direction	Control Vs. Case	Control Vs. Prodromal	Control Vs. LRRK2+	Control Vs. GBA+	Control Vs. SNCA+
**HLA-DQA2**	++	2.61×10^{-47}	++	*	***	***	NS	NS
**ITGB2**	++	4.49×10^{-47}	++	NS	***	***	NS	NS
**C1QTNF1**	++	6.96×10^{-46}	++	NS	***	***	NS	NS
**GRN**	++	4.32×10^{-42}	++	NS	***	**	NS	NS
**GPNMB (X8240.207)**	++	2.32×10^{-41}	++	NS	***	***	NS	NS
**GPNMB (X5080.131)**	++	1.48×10^{-38}	++	NS	***	***	NS	NS
**ENTPD1**	++	1.59×10^{-34}	++	NS	**	**	NS	NS
TMEM106A	++	1.51×10^{-32}	++	NS	***	NS	NS	NS
OLR1 (X3636.37)	--	0.576	NS	NS	*	NS	NS	NS
OLR1 (X7893.19)	--	0.717	NS	NS	*	NS	NS	NS
**GREM2**	++	NA	++	**	**	NS	NS	NS
**GAA**	++	NA	--	***	*	NS	NS	NS
**LCT**	--	NA	--	NS	**	**	*	NS
**LGALS9**	++	NA	++	NS	**	***	NS	NS
DNAJC15	--	NA	NS	NS	NS	NS	**	NS
CA1	--	NA	NS	NS	NS	NS	NS	NS
CD63	++	NA	NS	NS	NS	NS	NS	NS
CHIT1	--	NA	NS	NS	NS	NS	NS	NS
EID3	--	NA	NS	NS	NS	NS	NS	NS
FCGR1A	++	NA	NS	NS	NS	NS	NS	NS
FTL (X5934.1)	--	NA	NS	NS	NS	NS	NS	NS
GPNMB (X8606.39)	++	NA	NS	NS	NS	NS	NS	NS
NIPAL4	++	NA	NS	NS	NS	NS	NS	NS
SRI	--	NA	NS	NS	NS	NS	NS	NS

Direction of the top FDR corrected SNP, PWAS p-value, direction of PPMI protein levels, and violin plot significance of control vs. case, prodromal, and mutation (*LRRK2+*, *GBA+*, *SNCA+*) of the 21 proteins (24 aptamers) with PPMI cohort protein data. Proteins are sorted by PWAS p-value. Bolded proteins are control vs. *LRRK2+* and/or control vs. case significant. Only HLA-DQA2 is significant for both case Vs. control and case Vs. *LRRK2+*. PWAS p-value as NA refers to TWAS/FUSION excluding the gene from the analysis due to the aptamer's SNP-heritability p-value < 0.05. The significance level as stars with p-value equal to or less than 0.001 (***), 0.01 (**), and 0.05 (*). NS (not significant).

Supplementary Table 6 Sex differences in protein expression using PPMI cohort

	PPMI M vs F	Case M vs F	Control M vs F	Prodromal M vs F	LRRK2+ M vs F	GBA+ M vs F	SNCA+ M vs F
**# Male samples**	422	229	105	49	26	11	2
**# Female samples**	314	136	72	62	36	6	2
**Proteins**							
C1QTNF1	NS	NS	NS	NS	NS	NS	NS
CA1	NS	NS	NS	NS	NS	NS	NS
**CD63**	NS	*	NS	NS	NS	NS	NS
**CHIT1**	*	NS	NS	NS	NS	NS	NS
DNAJC15	NS	NS	NS	NS	NS	NS	NS
EID3	NS	NS	NS	NS	NS	NS	NS
ENTPD1	NS	NS	NS	NS	NS	NS	NS
<u>**FCGR1A**</u>	***	***	NS	NS	**	NS	NS
FTL (X5934.1)	NS	NS	NS	NS	NS	NS	NS
**GAA**	**	NS	*	NS	NS	NS	NS
GPNMB (X5080.131)	NS	NS	NS	NS	NS	NS	NS
GPNMB (X8240.207)	NS	NS	NS	NS	NS	NS	NS
<u>**GPNMB (X8606.39)**</u>	**	**	NS	NS	NS	NS	NS
**GREM2**	***	NS	NS	NS	NS	NS	NS
GRN	NS	NS	NS	NS	NS	NS	NS
HLA-DQA2	NS	NS	NS	NS	NS	NS	NS
ITGB2	NS	NS	NS	NS	NS	NS	NS
**LCT**	**	NS	**	NS	NS	NS	NS
**LGALS9**	*	NS	**	*	NS	NS	NS
NIPAL4	NS	NS	NS	NS	NS	NS	NS
**OLR1 (X3636.37)**	***	NS	NS	NS	NS	NS	NS
<u>**OLR1 (X7893.19)**</u>	***	**	NS	NS	NS	NS	NS
SRI	NS	NS	NS	NS	NS	NS	NS
**TMEM106A**	*	NS	NS	NS	NS	NS	NS

Violin plot significance of sex differences in protein expression for all samples with PPMI cohort protein data, cases, controls, and autosomal dominant PD mutations (*LRRK2+*, *GBA+*, *SNCA+*; prodromal cases) for the 21 proteins (24 aptamers). Bolded proteins are significant across PPMI, and the underlined proteins are also significant in case samples. The significance level as stars with p-value equal to or less than 0.001 (***), 0.01 (**), and 0.05 (*). NS (not significant).

Supplementary Table 7 SNPs in MR

Protein	Aptamer	MR SNPs RSID	Top SNP p-value	All SNP F-statistics	Excluding LRRK2 (chr12)	- LRRK2 F-statistics	Cis signal	Cis signal F-statistic
AGFG2	X23597.11	rs78951820, rs10764296	8.71×10^{-20}	82.977, 34.925	rs78951820, rs10764296	82.977, 34.925,	rs78951820	82.977
C1QTNF1	X6304.8	rs76904798, rs12465677	1.86×10^{-30}	131.231, 36.566	rs12465677	36.566	NA	NA
CD68	X20528.23	rs76904798, rs112958256, rs6976930, rs8417	4.37×10^{-63}	281.248, 41.308, 34.726, 34.126	rs112958256, rs6976930, rs8417	41.308, 34.726, 34.126	rs112958256	41.308
CD68	X18922.27	rs76904798, rs1293322, rs62466318, rs57372210	1.58×10^{-57}	254.915, 38.933, 35.720, 31.624	rs1293322, rs62466318, rs57372210	38.933, 35.720, 31.624	NA	NA
CHIT1	X3600.2	rs12141375, rs6857, rs7073430, rs8011695	3.47×10^{-175}	795.740, 43.928, 32.770, 31.242	rs12141375, rs6857, rs7073430, rs8011695	795.740, 43.928, 32.770, 31.242,	rs12141375	795.740
ENTPD1	X3182.38	rs76904798, rs13066753	3.72×10^{-29}	125.822, 33.368	rs13066753	33.368	NA	NA
GAA	X9385.4	rs2304850	2.24×10^{-10}	40.173	rs2304850	40.173,	rs2304850	40.173
GPNMB	X8240.207	rs858239, rs11564199, rs35277048	4.79×10^{-304}	1025.255, 145.948, 33.415	rs858239, rs35277048	1025.255, 33.415	rs858239	1025.255
GPNMB	X5080.131	rs858239, rs11564199, rs828266	6.76×10^{-225}	1385.785, 121.370, 29.842	rs858239, rs828266	1385.785, 29.842	rs858239	1385.785
GPNMB	X8606.39	rs858239	2.75×10^{-103}	466.448	rs858239	466.448	rs858239	466.448
GREM2	X5598.3	rs28903073, rs113411394	7.94×10^{-9}	33.348, 30.148	rs113411394	30.148	NA	NA
GRN	X4992.49	rs5848, rs76904798	1.26×10^{-20}	71.234, 86.966	rs5848	71.234	rs5848	71.234
HLA-DQA2	X7757.5	rs1846190, rs140722239	1.07×10^{-116}	529.174, 122.925	rs1846190	529.174	rs1846190	529.174
ITGB2	X12750.9	rs3135350, rs76904798, rs147816190	1.91×10^{-17}	72.428, 62.2298, 29.976	rs3135350, rs147816190	72.428, 29.976	NA	NA
LCT	X9017.58	rs3820794, rs550472465, rs924086	5.89×10^{-142}	644.810, 30.424, 30.360	rs3820794, rs550472465, rs924086	644.810, 30.424, 30.360	rs3820794	644.810
LGALS9	X9197.4	rs28425473, rs34593439	1.45×10^{-27}	118.541, 48.618	rs28425473, rs34593439	118.541, 48.618	rs28425473	118.541
OLR1	X3636.37	rs3816844, rs13066753	6.03×10^{-16}	65.216, 41.260	rs13066753	41.260	NA	NA
OLR1	X7893.19	rs3816844, rs13066753	4.79×10^{-15}	61.471, 39.113	rs13066753	39.113	NA	NA
SDCBP2	X19261.12	rs76904798	5.37×10^{-10}	38.564	NA	NA	NA	NA
TLR3	X16918.198	rs3775291, rs76904798	4.68×10^{-137}	620.247, 49.317	rs3775291	620.247	rs3775291	620.247
TMEM106A	X10499.1	rs76904798, rs35659126	2.57×10^{-17}	71.451, 37.793	rs35659126	37.793	NA	NA

Rs-id and F-statistics of the SNPs as instruments for each of the 21 aptamers (17 proteins) in MR. Cis signal SNPs are within ± 1 MB of the hg38 genome location for the gene encoding the protein.

Supplementary Table 8 Brain cell types of SomaScan7K and LRRK2 locus associated proteins

Brain Cell Type	SomaScan7K Proteins (>50% total expression)	25 Proteins (>50% total expression)	Enrichment (FC / p-value)
Neuron	525 (9.20%)	1 (4%)	-0.56 / 0.227
Microglia/Macrophage	418 (7.32%)	9 (36%)	**3.92 / 3.45 ×10^{-5}**
Endothelial	227 (3.98%)	1 (4%)	0.01 / 0.377
Mature Astrocyte	227 (3.98%)	3 (12%)	2.02 / 0.059
Oligodendrocyte	108 (1.89%)	2 (8%)	3.23 / 0.069
Non-specific	4204 (73.64%)	9 (36%)	-0.51 / **3.07 ×10^{-4}**

Unique proteins from CSF SomaScan7K with cell type data (N=5,709) and 25 LRRK2 associated proteins. The most common cell-type specific (>50% total expression) cell type in the CSF SomaScan7K was neuronal. The most common cell type of the 25 LRRK2 associated proteins was microglial/macrophage. Enrichment p-values by hypergeometric distribution using dhyper function in R.

Supplementary Table 9 Brain cell types of LRRK2 associated proteins

Gene	Max Cell Proportion	Max Cell Type
C1QTNF1	0.71	Endothelial
AGFG2	0.66	Mature astrocyte
SRI	0.59	Mature astrocyte
SDCBP2	0.58	Mature astrocyte
OLR1	0.93	Microglia/Macrophage
ITGB2	0.91	Microglia/Macrophage
CD68	0.91	Microglia/Macrophage
FCGR1A	0.87	Microglia/Macrophage
TMEM106A	0.85	Microglia/Macrophage
HLA-DQA2	0.83	Microglia/Macrophage
TLR3	0.79	Microglia/Macrophage
LGALS9	0.60	Microglia/Macrophage
GRN	0.56	Microglia/Macrophage
ENTPD1	0.49	Microglia/Macrophage
FTL	0.45	Microglia/Macrophage
GAA	0.42	Microglia/Macrophage
EID3	0.42	Microglia/Macrophage
GREM2	0.84	Neuron
NIPAL4	0.82	Oligodendrocyte
GPNMB	0.68	Oligodendrocyte
CD63	0.37	Mixed
DNAJC15	0.31	Mixed
CA1	0.22	Mixed
LCT	0.20	Mixed
CHIT1	0.20	Mixed

The sum column is the sum of the proportions of human mature astrocytes, neurons, microglia/macrophages, oligodendrocytes, and endothelial expression. Mixed cell type refers to either no cell type had a max proportion of expression (>40%) or all cell types had equal proportions. Genes are sorted by cell type and max cell proportion.

Supplementary References

1. Picca A, Guerra F, Calvani R, Marini F, Biancolillo A, Landi G, et al. Mitochondrial Signatures in Circulating Extracellular Vesicles of Older Adults with Parkinson's Disease: Results from the EXosomes in PArkiNson's Disease (EXPAND) Study. *J Clin Med*. 2020;9(2):504.
2. Garcia-Esparcia P, Hernández-Ortega K, Ansoleaga B, Carmona M, Ferrer I. Purine metabolism gene deregulation in Parkinson's disease. *Neuropathol Appl Neurobiol*. 2015;41(7):926-40.
3. Nalls MA, Blauwendraat C, Vallerga CL, et al. Identification of novel risk loci, causal insights, and heritable risk for Parkinson's disease: a meta-analysis of genome-wide association studies. *Lancet Neurol*. 2019;18(12):1091-1102.
4. Hill-Burns EM, Factor SA, Zabetian CP, Thomson G, Payami H. Evidence for more than one Parkinson's disease-associated variant within the HLA region. *PLoS One*. 2011;6(11):e27109.
5. Alcalay RN, Wolf P, Levy OA, Kang UJ, Waters C, Fahn S, et al. Alpha galactosidase A activity in Parkinson's disease. *Neurobiol Dis*. 2018;112:85-90.
6. Moloney EB, Moskites A, Ferrari EJ, Isacson O, Hallett PJ. The glycoprotein GPNMB is selectively elevated in the substantia nigra of Parkinson's disease patients and increases after lysosomal stress. *Neurobiol Dis*. 2018;120:1-11.
7. Genovese I, Giamogante F, Barazzuol L, Battista T, Fiorillo A, Vicario M, et al. Sorcin is an early marker of neurodegeneration, Ca2+dysregulation and endoplasmic reticulum stress associated to neurodegenerative diseases. *Cell Death Dis*. 2020;11(10):861.
8. Domenighetti C, Sugier PE, Ashok Kumar Sreelatha A, Schulte C, Grover S, Mohamed O, et al. Dairy Intake and Parkinson's Disease: A Mendelian Randomization Study. *Mov Disord*. 2022;37(4):857-864.
9. Xu E, Boddu R, Abdelmotilib HA, Kelly, K, Sokratian A Harms AS, et al. Pathologic α-Synuclein Species Activate LRRK2 in Pro-Inflammatory Monocyte and Macrophage Responses. *bioRxiv*. 2020.
10. George G, Singh S, Lokappa SB, Varkey J. Gene co-expression network analysis for identifying genetic markers in Parkinson's disease - a three-way comparative approach. *Genomics*. 2019;111(4):819-830.

11. Wang J, Liu Y, Liu Y, Zhu K, Xie A. The association between TLR3 rs3775290 polymorphism and sporadic Parkinson's disease in Chinese Han population. *Neurosci Lett*. 2020;728:135005.
12. Momcilovic O, Sivapatham R, Oron TR, Meyer M, Mooney S, Rao MS, et al. Derivation, Characterization, and Neural Differentiation of Integration-Free Induced Pluripotent Stem Cell Lines from Parkinson's Disease Patients Carrying SNCA, LRRK2, PARK2, and GBA Mutations. *PLoS One*. 2016 May 18;11(5):e0154890.
13. Go RCP, Corley MJ, Ross GW, Petrovitch H, Masaki KH, Maunakea AK, et al. Genome-wide epigenetic analyses in Japanese immigrant plantation workers with Parkinson's disease and exposure to organochlorines reveal possible involvement of glial genes and pathways involved in neurotoxicity. *BMC Neurosci*. 2020;21(1):31.
14. Fernandez MV, Budde JP, Eteleeb A, Wang F, Martinez R, Norton J, Gentsch J, et al. Functional exploration of AGFG2, a novel player in the pathology of Alzheimer disease. *Alzheimer's Dement*. 2021;17:e054240.
15. Mukherjee S, Klaus C, Pricop-Jeckstadt M, Miller JA, Struebing FL. A Microglial Signature Directing Human Aging and Neurodegeneration-Related Gene Networks. *Front Neurosci*. 2019;13:2.
16. Agarwal D, Sandor C, Volpato V, Caffrey TM, Monzón-Sandoval J, Bowden R, et al. A single-cell atlas of the human substantia nigra reveals cell-specific pathways associated with neurological disorders. *Nat Commun*. 2020;11(1):4183.
17. Cuttler K, Hassan M, Carr J, Cloete R, Bardien S. Emerging evidence implicating a role for neurexins in neurodegenerative and neuropsychiatric disorders. *Open Biol*. 2021;11(10):210091.
18. Forcella M, Lau P, Oldani M, Melchioretto P, Bogni A, Gribaldo L, et al. Neuronal specific and non-specific responses to cadmium possibly involved in neurodegeneration: A toxicogenomics study in a human neuronal cell model. *Neurotoxicology*. 2020;76:162-173.
19. Karayel O, Virreira Winter S, Padmanabhan S, Kuras YI, Vu DT, Tuncali I, et al. Proteome profiling of cerebrospinal fluid reveals biomarker candidates for Parkinson's disease. *Cell Rep Med*. 2022 Jun 21;3(6):100661.
20. Kurzawa-Akanbi M, Keogh M, Tsefou E, Ramsay L, Johnson M, Keers S, et al. Neuropathological and biochemical investigation of Hereditary Ferritinopathy cases with ferritin light chain mutation: Prominent protein aggregation in the absence of

major mitochondrial or oxidative stress. *Neuropathol Appl Neurobiol*. 2021;47(1):26-42.

21. Steelman AJ, Li J. Astrocyte galectin-9 potentiates microglial TNF secretion. *J Neuroinflammation*. 2014;11:144.
22. Dettori I, Fusco I, Bulli I, Gaviano L, Coppi E, Cherchi F, et al. Protective effects of carbonic anhydrase inhibition in brain ischaemia in vitro and in vivo models. *J Enzyme Inhib Med Chem*. 2021;36(1):964-976.
23. Satoh J, Kino Y, Kawana N, Yamamoto Y, Ishida T, Saito Y, et al. TMEM106B expression is reduced in Alzheimer's disease brains. *Alzheimers Res Ther*. 2014;6(2):17.
24. Laadhar S, Ben Mansour R, Marrakchi S, Miled N, Ennouri M, Fischer J, et al. Identification of a novel missense mutation in NIPAL4 gene: First 3D model construction predicted its pathogenicity. *Mol Genet Genomic Med*. 2020;8(3):e1104.
25. Juanes-Velasco P, Galicia N, Pin E, Jara-Acevedo R, Carabias-Sánchez J, García-Valiente R, et al. Deciphering Biomarkers for Leptomeningeal Metastasis in Malignant Hemopathies (Lymphoma/Leukemia) Patients by Comprehensive Multipronged Proteomics Characterization of Cerebrospinal Fluid. *Cancers (Basel)*. 2022;14(2):449.

Supplement 2: Dominantly Inherited Alzheimer Network (DIAN) consortia investigators and coordinators

Name	Affiliation	Email address
Sarah Adams	Washington University in St. Louis School of Medicine	sladams@wustl.edu
Ricardo Allegri	Institute of Neurological Research Fleni, Buenos Aires, Argentina	rallegri@fleni.org.ar
Aki Araki	Niigata University	araki-aki@bri.niigata-u.ac.jp
Nicolas Barthelemy	Washington University in St. Louis School of Medicine	barthelemy.nicolas@wustl.edu
Randall Bateman	Washington University in St. Louis School of Medicine	batemanr@wustl.edu
Jacob Bechara	Neuroscience Research Australia	j.bechara@neura.edu.au
Tammie Benzinger	Washington University in St. Louis School of Medicine	benzingert@wustl.edu
Sarah Berman	University of Pittsburgh	bermans@upmc.edu
Courtney Bodge	Brown University-Butler Hospital	Cbodge@Butler.org
Susan Brandon	Washington University in St. Louis School of Medicine	brandons@wustl.edu
William (Bill) Brooks	Neuroscience Research Australia	w.brooks@NeuRA.edu.au
Jared Brosch	Indiana University	
Jill Buck	Indiana University	jilmbuck@iu.edu
Virginia Buckles	Washington University in St. Louis School of Medicine	bucklesv@wustl.edu
Kathleen Carter	Emory University School of Medicine	emma.kathleen.carter@emory.edu
Lisa Cash	Washington University in St. Louis School of Medicine	cashl@wustl.edu
Charlie Chen	Washington University in St. Louis School of Medicine	chenc@wustl.edu
Jasmeer Chhatwal	Brigham and Women's Hospital–Massachusetts General Hospital	Chhatwal.Jasmeer@mgh.harvard.edu
Patricio Chrem	Institute of Neurological Research Fleni, Buenos Aires, Argentina	pchremmendez@fleni.org.ar
Jasmin Chua	Washington University in St. Louis School of Medicine	chuajk@wustl.edu
Helena Chui	University of Southern California	helena.chui@med.usc.edu
Carlos Cruchaga	Washington University in St. Louis School of Medicine	cruchagac@wustl.edu
Gregory S Day	Mayo Clinic Jacksonville	Day.gregory@mayo.edu
Chrismary De La Cruz	Columbia University	
Darcy Denner	Washington University in St. Louis School of Medicine	drdenner@wustl.edu
Anna Diffenbacher	German Center for Neurodegenerative Diseases (DZNE) Munich	Anna.dieffenbacher@dzne.de
Aylin Dincer	Washington University in St. Louis School of Medicine	aylin.dincer@wustl.edu
Tamara Donahue	Washington University in St. Louis School of Medicine	tammie@wustl.edu
Jane Douglas	University College London	jane.douglas@ucl.ac.uk
Duc Duong	Emory University School of Medicine	dduong@emory.edu
Noelia Egido	Institute of Neurological Research Fleni, Buenos Aires, Argentina	negido@fleni.org.ar
Bianca Esposito	Icahn School of Medicine at Mount Sinai	bianca.esposito@mssm.edu
Anne Fagan	Washington University in St. Louis School of Medicine	fanana@wustl.edu
Marty Farlow	Indiana University	mfarlow@iupui.edu
Becca Feldman	Washington University in St. Louis School of Medicine	rebeccaf@wustl.edu
Colleen Fitzpatrick	Brigham and Women's Hospital-Massachusetts	cdfitzpatrick@bwh.harvard.edu
Shaney Flores	Washington University in St. Louis School of Medicine	sflores@wustl.edu
Nick Fox	University College London	n.fox@ucl.ac.uk
Erin Franklin	Washington University in St. Louis School of Medicine	efranklin@wustl.edu
Nelly Friedrichsen	Washington University in St. Louis School of Medicine	n.joseph@wustl.edu
Hisako Fujii	Osaka City University	hfujii@med.osaka-cu.ac.jp

Samantha Gardener	Edith Cowan University, Perth	s.gardener@ecu.edu.au
Bernardino Ghetti	Indiana University	bghetti@iupui.edu
Alison Goate	Icahn School of Medicine at Mount Sinai	alison.goate@mssm.edu
Sarah Goldberg	University of Pittsburgh	goldbergs2@upmc.edu
Jill Goldman	Columbia University	JG2673@cumc.columbia.edu
Alyssa Gonzalez	Washington University in St. Louis School of Medicine	alyssa.gonzales@wustl.edu
Brian Gordon	Washington University in St. Louis School of Medicine	bagordon@wustl.edu
Susanne Gräber-Sultan	DZNE-Tübingen	susanne.graeber-sultan@dzne.de @dzne.de
Neill Graff-Radford	Mayo Clinic Jacksonville	graffradford.neill@mayo.edu
Morgan Graham	Mayo Clinic Jacksonville	Graham.Morgan@mayo.edu
Julia Gray	Washington University in St. Louis School of Medicine	gray@wustl.edu
Emily Gremminger	Washington University in St. Louis School of Medicine	egremminger@wustl.edu
Miguel Grilo	University College London	m.grilo@ucl.ac.uk
Alex Groves	Washington University in St. Louis School of Medicine	amgroves@wustl.edu
Christian Haass	Ludwig-Maximilians University - Munich	Christian.Haass@mail03.med.uni-muenchen.de
Lisa Häsler	German Center for Neurodegenerative Diseases (DZNE), Tübingen	Lisa.Haesler@dzne.de
Jason Hassenstab	Washington University in St. Louis School of Medicine	hassenstabj@wustl.edu
Cortaiga Hellm	Washington University in St. Louis School of Medicine	cortaiga.hellm@wustl.edu
Elizabeth Herries	Washington University in St. Louis School of Medicine	e.herries@wustl.edu
Laura Hoechst-Swisher	Washington University in St. Louis School of Medicine	goodl@wustl.edu
Anna Hofmann	German Center for Neurodegenerative Diseases (DZNE), Tübingen	Anna.Hofmann@med.uni-tuebingen.de
David Holtzman	Washington University in St. Louis School of Medicine	holtzman@wustl.edu
Russ Hornbeck	Washington University in St. Louis School of Medicine	russ@wustl.edu
Yakushev Igor	German Center for Neurodegnerative Diseases (DZNE) Munich	Igor.yakushev@tum.de
Ryoko Ihara	Tokyo University	ihara-tky@umin.ac.jp
Takeshi Ikeuchi	Niigata University	ikeuchi@bri.niigata-u.ac.jp
Snezana Ikonomovic	University of Pittsburgh	ikonomovics@upmc.edu
Kenji Ishii	Niigata University/Tokyo University	ishii@pet.tmig.or.jp
Clifford Jack	Mayo Clinic Rochester	jack.clifford@mayo.edu
Gina Jerome	Washington University in St. Louis School of Medicine	ginajerome@wustl.edu
Erik Johnson	Emory University School of Medicine	erik.johnson@emory.edu
Mathias Jucker	German Center for Neurodegenerative Diseases (DZNE), Tübingen	mathias.jucker@uni-tuebingen.de
Celeste Karch	Washington University in St. Louis School of Medicine	karchc@wustl.edu
Stephan Käser	German Center for Neurodegenerative Diseases (DZNE), Tübingen	Stephan.kaeser@uni-tuebingen.de
Kensaku Kasuga	Niigata University	ken39@bri.niigata-u.ac.jp
Sarah Keefe	Washington University in St. Louis School of Medicine	sarahkeefe@wustl.edu
William (Bill) Klunk	University of Pittsburgh	klunkwe@gmail.com
Robert Koeppe	University of Michigan	koeppe@umich.edu
Deb Koudelis	Washington University in St. Louis School of Medicine	delanod@wustl.edu
Elke Kuder-Buletta	German Center for Neurodegenerative Diseases (DZNE), Tübingen	elke.kuder-buletta@dzne.de
Christoph Laske	German Center for Neurodegenerative Diseases (DZNE), Tübingen	christoph.laske@med.uni-tuebingen.de
Allan Levey	Emory University School of Medicine	alevey@emory.edu

Johannes Levin	German Center for Neurodegnerative Diseases (DZNE) Munich	Johannes.Levin@med.uni-muenchen.de
Yan Li	Washington University in St. Louis School of Medicine	yanli833@wustl.edu
Oscar Lopez	University of Pittsburgh	lopezol@upmc.edu
Jacob Marsh	Washington University in St. Louis School of Medicine	jacobmarsh@wustl.edu
Rita Martinez	Washington University in St. Louis School of Medicine	Ritamartinez@wustl.edu
Ralph Martins	Edith Cowan University	r.martins@ecu.edu.au
Neal Scott Mason	University of Pittsburgh Medical Center	masonss@upmc.edu
Colin Masters	University of Melbourne	c.masters@unimelb.edu.au
Kwasi Mawuenyega	Washington University in St. Louis School of Medicine	mawuenyegak@wustl.edu
Austin McCullough	Washington University in St. Louis School of Medicine	amccullough@wustl.edu
Eric McDade	Washington University in St. Louis School of Medicine	ericmcdade@wustl.edu
Arlene Mejia	Columbia University	am4717@cumc.columbia.edu
Estrella Morenas-Rodriguez	Ludwig-Maximilians University, Munich	Estrella.Morenas-Rodriguez@dzne.d
John Morris	Washington University in St. Louis School of Medicine	jcmorris@wustl.edu
James MountzMD	University of Pittsburgh	mountzjm@upmc.edu
Cath Mummery	University College London	c.mummery@ucl.ac.uk
Neelesh Nadkarni	University of Pittsburgh	nadkarnink@upmc.edu
Akemi Nagamatsu	Tokyo University	mail:akm77-tky@umin.ac.jp
Katie Neimeyer	Columbia University	kn2416@cumc.columbia.edu
Yoshiki Niimi	Tokyo University	niimiy-crc@h.u-tokyo.ac.jp
James Noble	Columbia University	jn2054@columbia.edu
Joanne Norton	Washington University in St. Louis School of Medicine	nortonj@wustl.edu
Brigitte Nuscher	Ludwig-Maximilians University, Munich	Brigitte.Nuscher@mail03.med.uni-muenchen.de
Antoinette O'Connor	University College London	antoinette.o'connor@ucl.ac.uk
Ulricke Obermüller	Hertie Institute for Clinical Brain Research	ulrike.obermueller@klinikum.uni-tuebingen.de
Riddhi Patira	University of Pittsburgh	patirar@upmc.edu
Richard Perrin	Washington University in St. Louis School of Medicine	rperrin@wustl.edu
Lingyan Ping	Emory University School of Medicine	lingyan.ping@emory.edu
Oliver Preische	German Center for Neurodegenerative Diseases (DZNE), Tübingen	Oliver.Preische@med.uni-tuebingen.de
Alan Renton	Icahn School of Medicine at Mount Sinai	alan.renton@mssm.edu
John Ringman	University of Southern California	john.ringman@med.usc.edu
Stephen Salloway	Brown University-Butler Hospital	SSalloway@Butler.org
Peter Schofield	Neuroscience Research Australia	p.schofield@neura.edu.au
Michio Senda	Osaka City University	michio_senda@kcho.jp
Nick Seyfried	Emory University School of Medicine	nseyfri@emory.edu
Kristine Shady	Washington University in St. Louis School of Medicine	kesh238@g.uky.edu
Hiroyuki Shimada	Osaka City University	h.shimada@med.osaka-cu.ac.jp
Wendy Sigurdson	Washington University in St. Louis School of Medicine	sigurdsonw@wustl.edu
Jennifer Smith	Washington University in St. Louis School of Medicine	smith.jennifer@wustl.edu
Lori Smith	University of Pittsburgh	macedonials@upmc.edu
Beth Snitz	University of Pittsburgh	snitbe@upmc.edu
Hamid Sohrabi	Edith Cowan University	h.sohrabi@ecu.edu.au
Sochenda Stephens	Mayo Clinic Jacksonville	Stephens.Sochenda@mayo.edu

Kevin Taddei	Edith Cowan University	k.taddei@ecu.edu.au
Sarah Thompson	University of Pittsburgh	thompsons24@upmc.edu
Jonathan Vöglein	German Center for Neurodegnerative Diseases (DZNE) Munich	Jonathan.voeglein@med.uni-muenchen.de
Peter Wang	Washington University in St. Louis School of Medicine	guoqiao@wustl.edu
Qing Wang	Washington University in St. Louis School of Medicine	wangqing@wustl.edu
Elise Weamer	University of Pittsburgh	weamerea@upmc.edu
Chengjie Xiong	Washington University in St. Louis School of Medicine	chengjie@wustl.edu
Jinbin Xu	Washington University in St. Louis School of Medicine	jinbinxu@wustl.edu
Xiong Xu	Washington University in St. Louis School of Medicine	xxu@wustl.edu

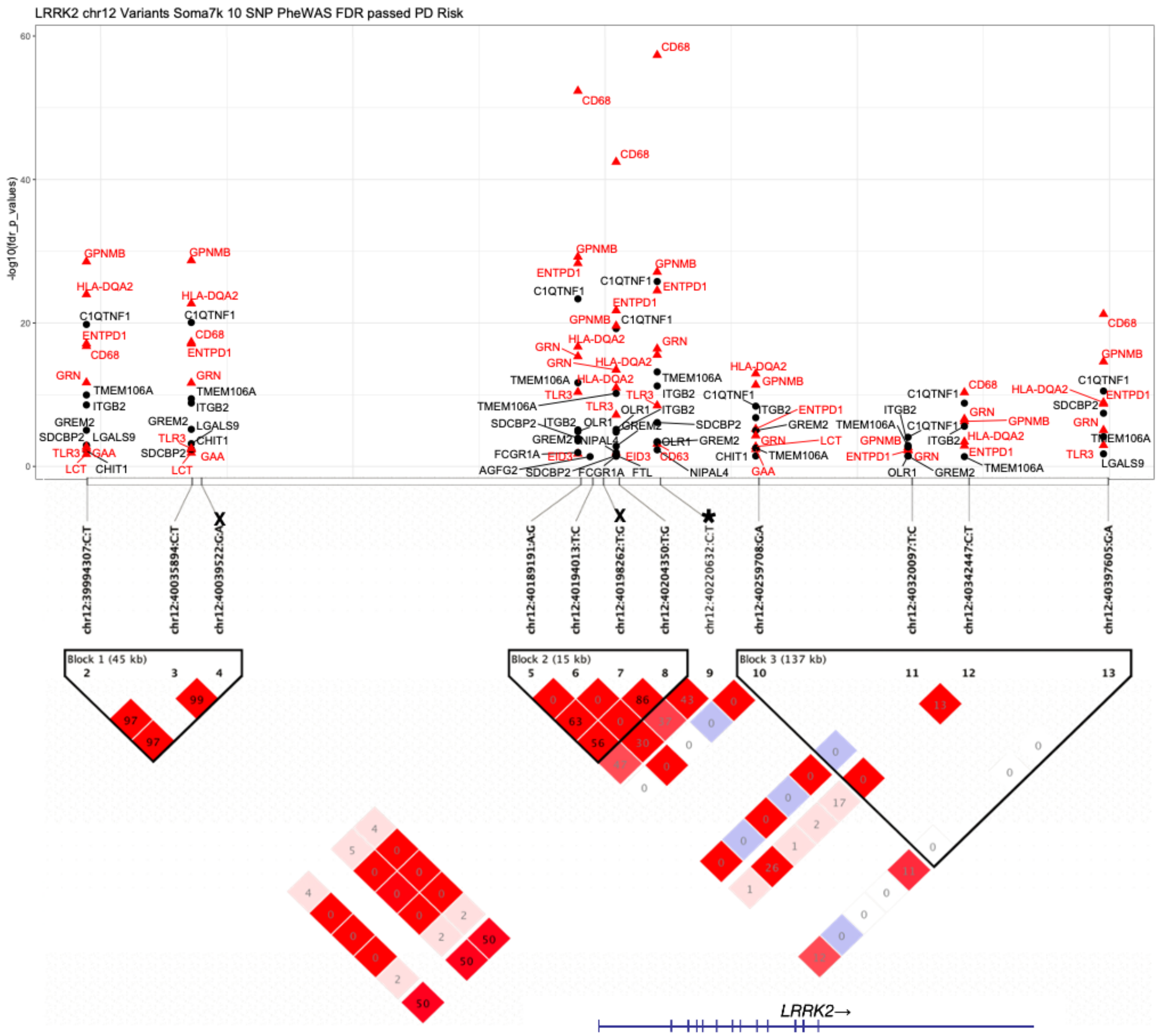

Supplementary Figure 1: PheWAS and LD matrix of *LRRK2* variants. PheWAS plot of 10 SNPs in chr12 LRRK2. FDR corrected -log10(p-value) y-axis. 25 unique CSF proteins pass FDR with 11 having known PD risk association (red triangles). LD plot of 12 independently associated Chr12 SNPS, 10 are present with chr12:40220632:C:T (*) and $r^2 >$ 0.85 per LD block removed (X). The 5 independently associated SNPs within the *LRRK2* region (Chr12:40,196,744-40,369,285) is below the LD plot with a blue line.

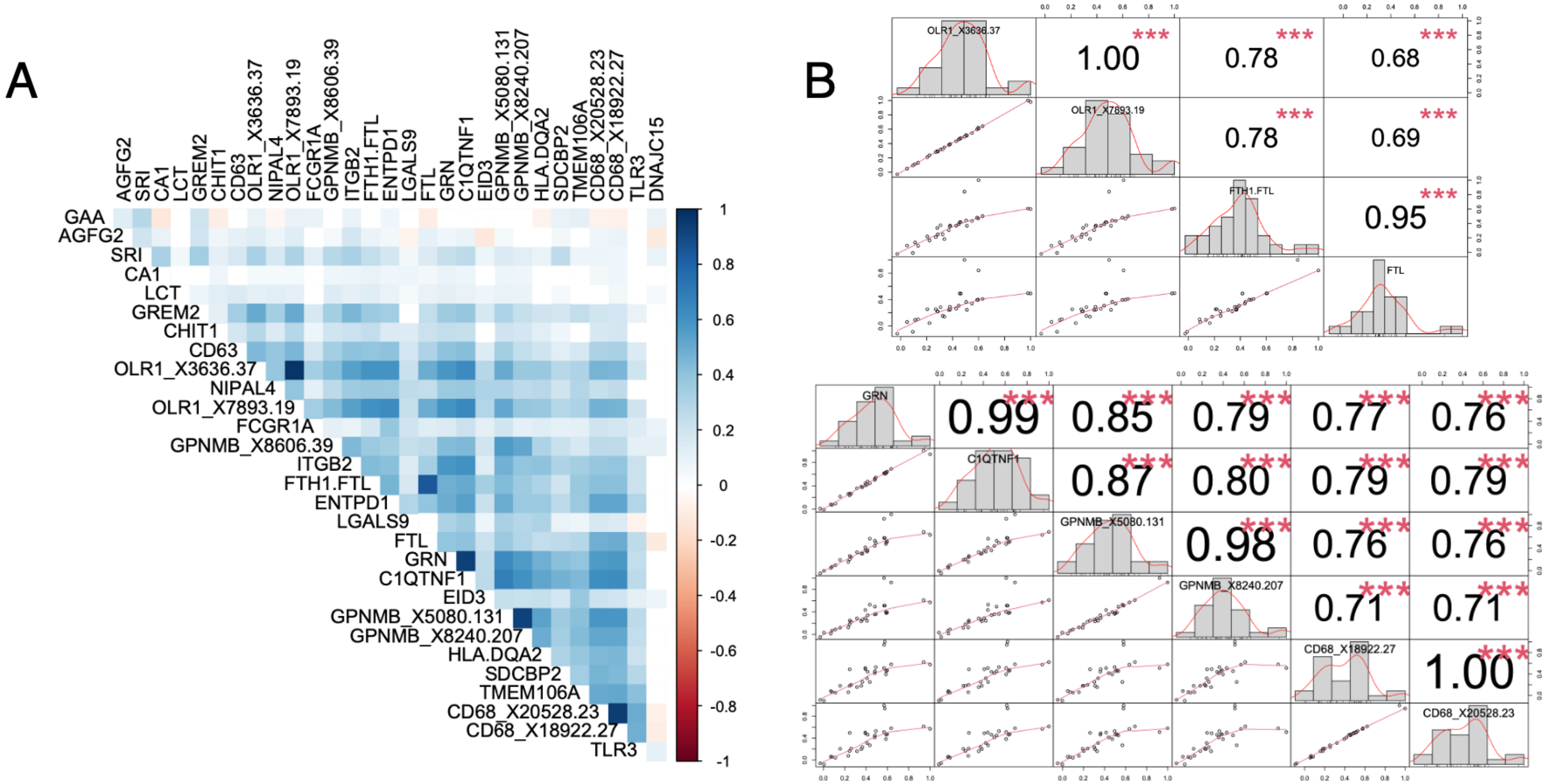

Supplementary Figure 2: Protein-protein correlation. (A) Pearson correlation coefficient matrix of the 25 proteins. (B) Chart correlation of the significant regions of OLR1 and FTL aptamers above and GRN below. The distribution of each protein is shown on the diagonal with the bottom displaying the bivariate scatterplots with a fitted line. The top of the diagonal shows the correlation value and the significance level as stars with p-value equal to or less than 0.001 (***), 0.01 (**), and 0.05 (*).

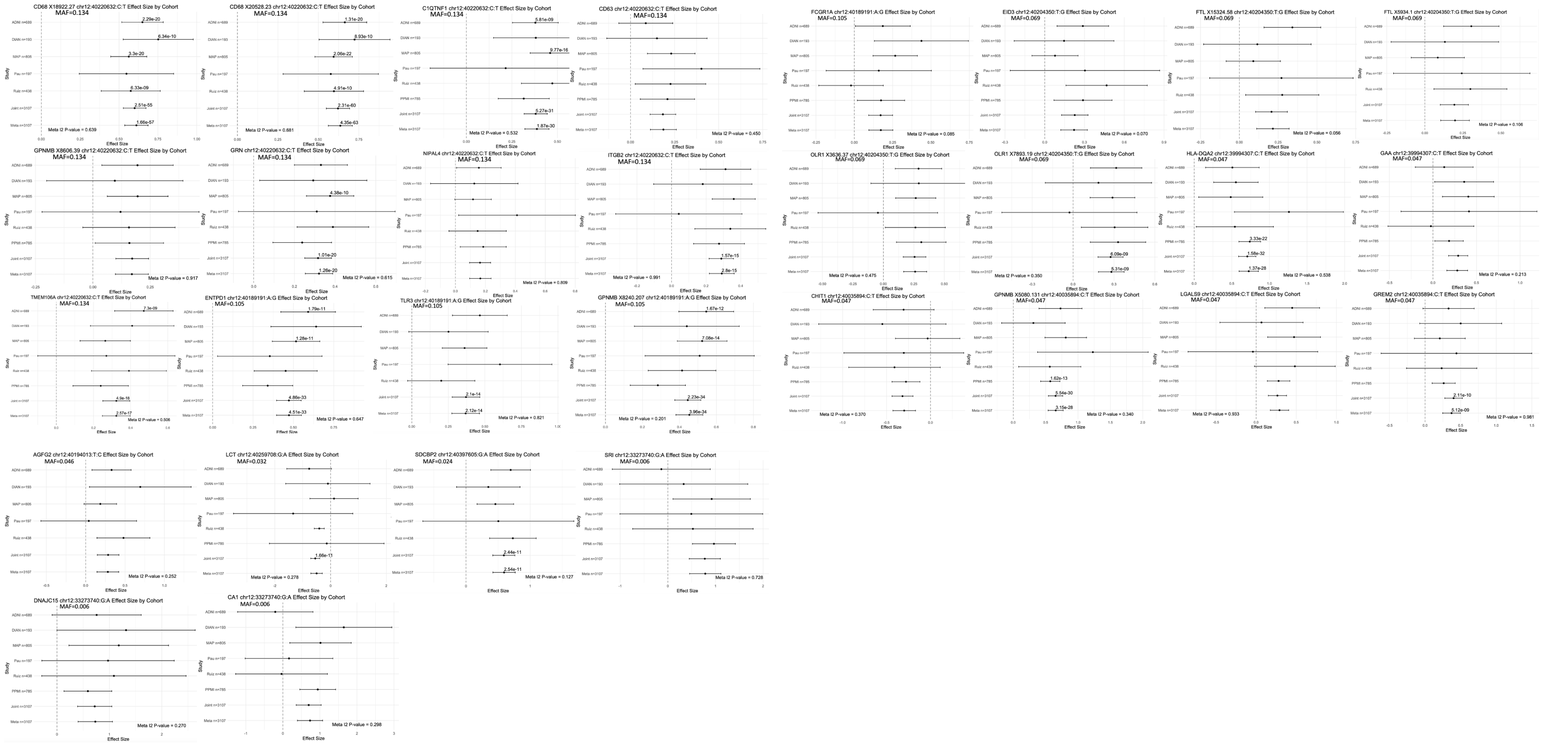

Supplementary Figure 3: Forest plots of the independent datasets. Forest plots of the effect sizes of signals in each of the 25 proteins (30 aptamers) for each independent dataset, the meta pQTL analysis, and the joint analysis. There were 21 proteins (24 aptamers) with PPMI information. Genome-wide significant p-values are shown.

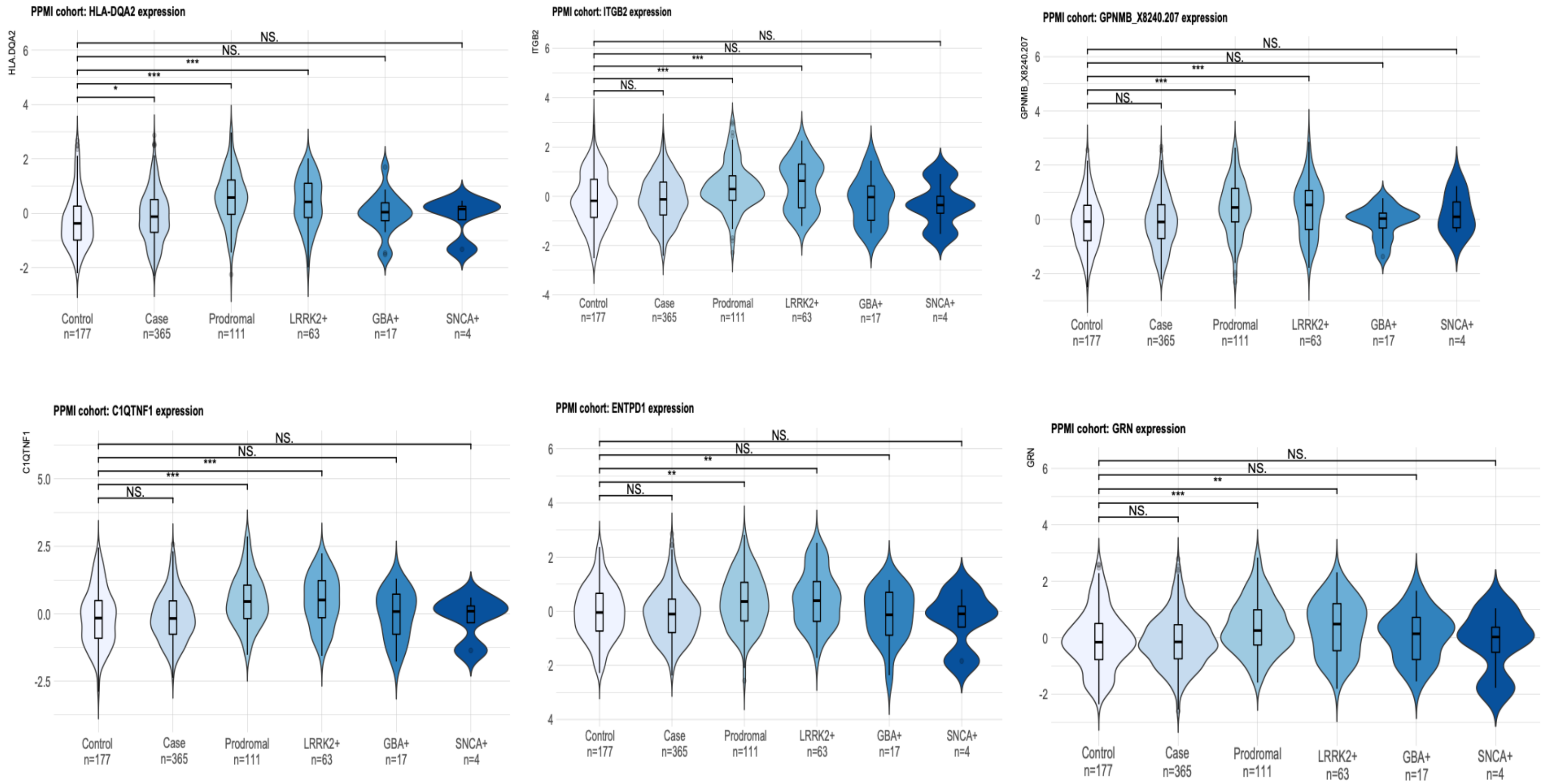

Supplementary Figure 4: PPMI significant PWAS Violin plots. Plot of *HLA-DQA2*, *C1QTNF1*, *ITGB2, ENTPD1, GPNMB*, & *GRN* gene expression. Control Vs. PD case and mutation carriers (*LRRK2*$^+$*, GBA*$^+$*,* and *SNCA*$^+$) (light to dark blue). The significance level as stars with p-value equal to or less than 0.001 (***), 0.01 (**), and 0.05 (*).

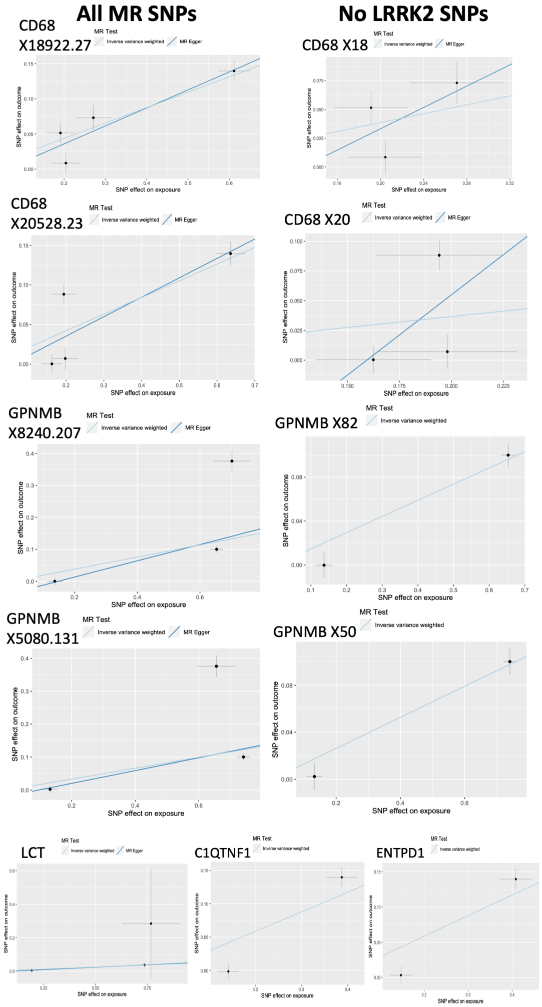

Supplementary Figure 5: Representative MR plots. Scatter plots of effect of SNP on exposure (pQTL) compared to effect of SNP on outcome (PD GWAS) in the Mendelian randomization analyses. The slope of the lines is the estimated causal effect for each MR test. The MR scatter plots require at least two SNPs and significant proteins in MR with at least two SNPs are shown. All MR SNP plots are on the left column and the no LRRK2 SNP MR is on the right. LCT has no LRRK2 SNPs in the MR and C1QTNF1 and ENTPD1 had two SNPs in the all MR SNP plots and there were not enough SNPs to plot the no LRRK2 MR.

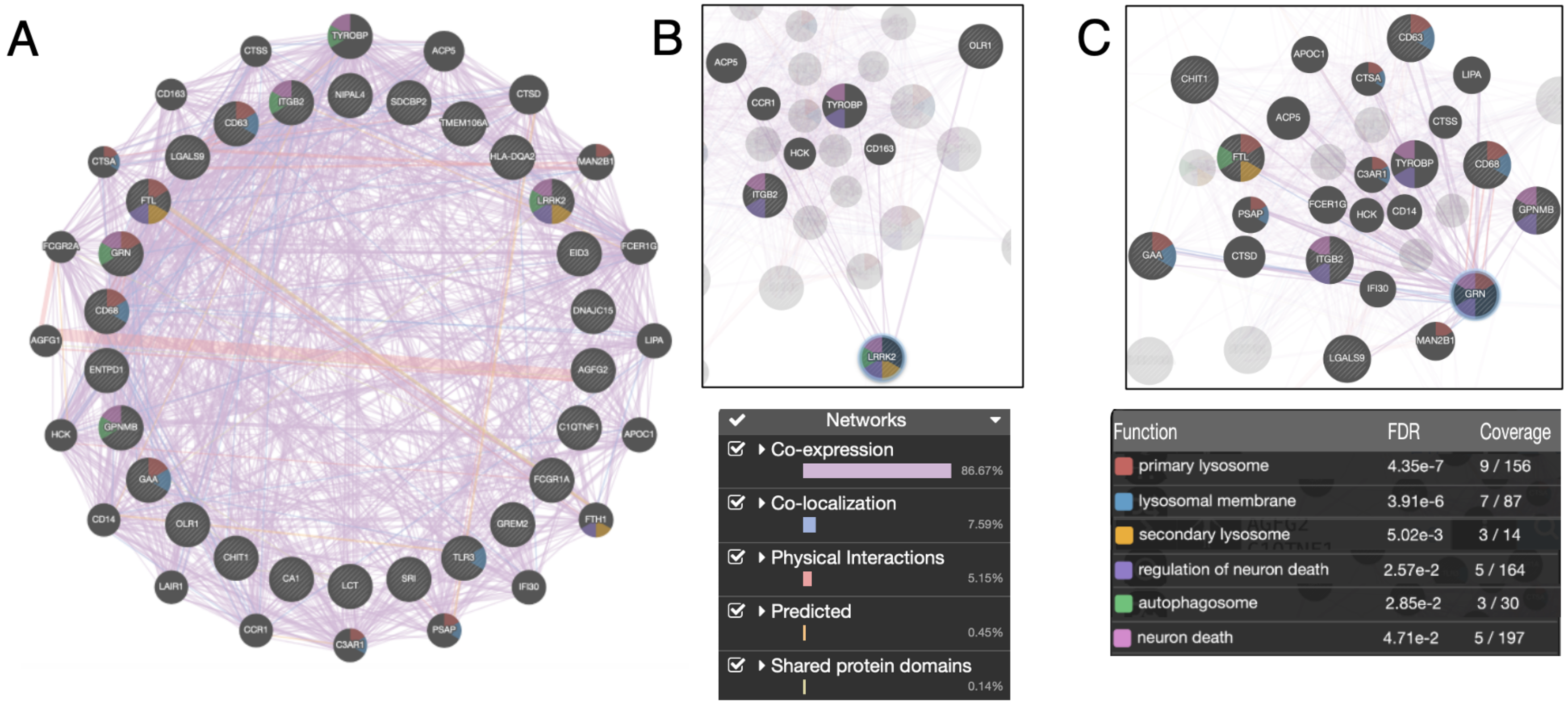

Supplementary Figure 6: Interaction pathways of LRRK2 associated proteins. (A) GeneMANIA identified 20 affiliated genes with network interaction and pathways of the 25 genes and *LRRK2*. (B) LRRK2 interactions. (C) GRN & GPNMB interactions.
